# Smoking and Quitting Behaviors by Different Indicators of Socioeconomic Position in England: A Population Study, 2014 to 2023

**DOI:** 10.1093/ntr/ntag003

**Published:** 2026-02-11

**Authors:** Annika Theodoulou, Jamie Hartmann-Boyce, Nicola Lindson, Thomas R Fanshawe, Sarah E Jackson

**Affiliations:** Nuffield Department of Primary Care Health Sciences, University of Oxford, Oxford, UK; Department of Health Promotion and Policy, University of Massachusetts, Amherst, MA, USA; Nuffield Department of Primary Care Health Sciences, University of Oxford, Oxford, UK; Nuffield Department of Primary Care Health Sciences, University of Oxford, Oxford, UK; Department of Behavioural Science and Health, University College London, London, UK

## Abstract

**Introduction:**

The social gradient in smoking is well-established but how associations differ across different indicators of socioeconomic position (SEP) is less clear. This study aimed to estimate associations of five SEP indicators with smoking and quitting-related behaviors.

**Methods:**

We used nationally representative, cross-sectional survey data on 195 543 adults aged ≥18 years in England surveyed between January, 2014 and December, 2023. Exposures were occupational social grade, employment status, housing tenure, educational-level, and household income. Outcomes were smoking prevalence; motivation to stop smoking; level of tobacco addiction and past-year quit attempts; and use of cessation aids and quitting success. Associations between exposure and outcome variables were estimated using logistic or linear regression, as appropriate. Covariates included age, gender, survey year, and (for the latter two outcomes) level of tobacco addiction.

**Results:**

Across all measures of SEP, increasing disadvantage was associated with higher odds of smoking, higher levels of tobacco addiction, and lower motivation to stop smoking. People from more disadvantaged occupational social grades, on lower household incomes and with less education were less likely to have made a quit attempt in the past year relative to those in the most advantaged categories. Odds of quitting success were lower in more disadvantaged housing tenures; however no clear evidence was observed when looking at other indicators of SEP. There were some differences in use of e-cigarettes as a cessation aid by SEP, but these either varied greatly across different SEP indicators or were inconclusive. There was a lack of clear evidence for all other cessation aids.

**Conclusions:**

Inequalities in smoking persist across multiple forms and degrees of disadvantage across England.

**Implications:**

Findings suggest that research investigating smoking prevalence, level of tobacco addiction, motivation to stop smoking, quit attempts, and quitting success may not need to assess multiple indicators of socioeconomic position when exploring relationships between these outcomes and disadvantaged groups, as results are similar regardless of the measure used to assess socioeconomic position. However, this does not necessarily imply that tailoring smoking cessation interventions for people with, for example, less education or on less income, will equivalently address socioeconomic inequalities in smoking, or that one type of tailored intervention will be similar in effectiveness across different forms, or degree, of disadvantage.

## Background

Tobacco use remains a leading cause of preventable morbidity and mortality worldwide. In England, official estimates suggest 11.9% of adults smoke cigarettes, but rates are significantly higher among people who are socioeconomically disadvantaged (e.g. those who are unemployed or have lower levels of education).^1^ Greater smoking prevalence translates to disproportionate tobacco-related disease, disability, and premature death in disadvantaged groups.^2^ This social gradient in smoking rates across England has also been observed more widely across the United Kingdom,^3^ and in other higher-income countries.^4^^,^^5^

In 2019, the UK government set an ambition to reach Smokefree 2030; that is, to reduce smoking rates to ≤5.0% in England by 2030.^6^ A recent independent review suggested that without intervention, England will not reach this target until at least 2037, while the poorest areas will lag even further behind and not meet this goal until 2044.^7^

Higher smoking rates among disadvantaged groups may arise due to higher rates of smoking uptake and/or lower rates of sustained smoking cessation. While people from more disadvantaged socioeconomic positions are more likely to initiate smoking,^8^ some evidence suggests that these groups also experience lower rates of quit success despite equivalent attempts to quit.^9^ This highlights that prevention is not enough, and targeted and evidence-based methods must be employed to support all 6 million people in the United Kingdom to quit smoking.^1^

Socioeconomic position is a complex construct which is context dependent, and can impact health in many ways.^10^^,^^11^ It can be operationalized through a multitude of indicators measured at an individual or area-based level, with each emphasizing a specific aspect of social stratification.^10^^,^^11^ Although these socioeconomic indicators tend to be highly correlated, there may be no single “best” indicator when investigating health outcomes.^12^

To develop effective interventions to reduce socioeconomic inequalities in smoking, we need to better understand current relationships between socioeconomic position, and smoking, and quitting behaviors. More specifically, we need to explore how smoking prevalence, levels of tobacco addiction, motivation to quit, quit attempts, use of evidence-based cessation aids and quitting success differ by socioeconomic position, not only overall, but also across different indicators. This will allow identification of potential differences in these relationships across various forms of disadvantage.

A cross-sectional survey carried out in England between 2015 and 2020 investigated smoking patterns by housing tenure; one of many measures of socioeconomic position.^13^ Motivation to quit was similar across people living in different housing types. However, people living in social housing (renting from local authority or housing association) had higher nicotine dependence, were more likely to make a quit attempt and use cessation support within the previous year, and had lower success in quitting than people living in other housing tenures (e.g. owned outright).^13^ It is not clear whether such relationships would be consistent if disadvantage was measured using a different indicator of socioeconomic position. In addition, the effectiveness of interventions tailored to reduce socioeconomic inequalities in smoking may perform differently across different types of disadvantaged groups.

### Aims and Research Questions

This study aimed to investigate associations between a range of different indicators of socioeconomic position and smoking and quitting behaviors. Using cross-sectional data from a large, nationally-representative population survey in England collected over a 10-year period, we addressed the following research questions using five distinct measures of socioeconomic position (occupational social grade, employment status, housing tenure, education level, and household income):

1. Among adults in England, to what extent does smoking prevalence differ by different indicators of socioeconomic position after adjusting for age, gender, and survey year?
2. Among people who currently smoke, to what extent does motivation to stop smoking differ by different indicators of socioeconomic position after adjusting for age, gender, and survey year?
3. Among past-year smokers, to what extent does the level of tobacco addiction differ by different indicators of socioeconomic position after adjusting for age, gender, and survey year?
4. Among past-year smokers, to what extent does the rate of past-year quit attempts differ by different indicators of socioeconomic position after adjusting for age, gender, and survey year?
5. Among past-year smokers who made a quit attempt, to what extent does the use of cessation aids and quitting success differ by different indicators of socioeconomic position after adjusting for age, gender, survey year, and level of tobacco addiction?

## Methods

Full details of the methods, including survey response options, are provided in the study protocol.^14^

### Study Design and Population

We used data from the Smoking Toolkit Study (STS), an ongoing, cross-sectional survey of a representative sample of adults in England.^15^ Every month, a hybrid of random probability and simple quota sampling is used to recruit a new sample of ~1700 adults aged ≥16 years. Comparisons with other national surveys and sales data have indicated that key variables such as sociodemographic characteristics and smoking prevalence are nationally representative.^15^^,^^16^

For the present study, we used data from respondents aged ≥18 years who completed the survey between January 2014 and December 2023. We selected subsamples based on participants’ smoking status to examine each research question (see Supplementary Table S1).

### Measures

#### Exposure Variables

***Occupational social grade*** was measured using the British National Readership Survey (NRS) Social-Grade Classification Tool,^17^ which categorizes respondents as: AB (higher managerial, administrative or professional); C1 (supervisory or clerical and junior managerial, administrative or professional); C2 (skilled manual workers); D (semi-skilled and unskilled manual workers); E (casual or lowest grade workers, pensioners, and others who depend on the welfare state for their income).

***Employment status*** was categorized as: paid work; student; not in paid work; retired.

***Housing tenure*** was categorized as: owner occupied; private rented (renting from private landlord); social rented (rented from local authority/housing association); other.

***Educational level*** was categorized as: university degree; A-level or equivalent (high school senior); General Certificate of Secondary Education (GCSE)/O-level/CSE (high school sophomore) or vocational qualification (high school senior); no post-16 formal qualifications; other or still studying.

***Annual household income*** was categorized as: £50 000+; £25 000–49 999; £13 500–24 999; up to £13 499. Data for this variable were not collected from March 2020 onwards.

#### Outcome Variables

***Smoking prevalence** (assessed in all respondents)* was categorized as “currently smoking” or “not currently smoking” (Supplementary Table S1).

***Motivation to stop smoking** (assessed in current smokers)* was assessed with the Motivation to Stop Scale,^18^ with scores ranging from 1 (“I don’t want to stop smoking”) to 7 (“I REALLY want to stop smoking and intend to in the next month”).

***Level of tobacco addiction** (assessed in past-year smokers)* was categorized based on self-rated strength of urges to smoke in the past 24 hours, with scores ranging from 0 (“not at all”) to 5 (“extremely strong”).^19^

***Quit attempts***  *(assessed in past-year smokers)* were categorized as 0 vs. ≥1 serious attempt to quit in the last year.

***Use of different cessation aids***  *(assessed in past-year smokers who made a serious quit attempt)* was categorized as use vs. non-use of the following aids (each analyzed as a separate variable): over the counter nicotine replacement therapy (NRT); electronic cigarettes; prescription medications (prescription NRT, varenicline, and bupropion); face-to-face behavioral support; prescription NRT; bupropion; varenicline; telephone support; self-help; and alternative treatments. Given the large number of analyses, we present results for some of these in supplementary materials.

***Quit success***  *(assessed in past-year smokers who made a serious quit attempt)* was assessed by asking “How long did your most recent serious quit attempt last before you went back to smoking?” Responses were categorized as still not smoking (ie quit success) vs. other responses.

#### Covariates

All analyses were adjusted for age, gender, and survey year. Age was categorized into age brackets of 18–24, 25–34, 35–44, 45–54, 55–64, and 65+ years, to account for the non-linear association between age and tobacco smoking. Gender was self-reported as “male,” “female” or “other” (the latter was excluded from analysis due to small sample size). Analyses of quit success and quit attempts were also adjusted for level of tobacco addiction (as described above).

### Data Analysis

The data analysis plan was preregistered on Open Science Framework and analyses were conducted using R v.4.3.2.^14^

We aggregated data from monthly surveys over a 10-year period (2014–2023). Data for estimates of prevalence were weighted using a rim (marginal) weighting technique to match the English population profile, relevant to the time each monthly survey was taken.^15^ Weighted and unweighted data were used for all estimates of association, with unweighted results reported in supplementary tables. Descriptive statistics were tabulated for outcome and exposure variables. We graphically presented annual trends in smoking prevalence over time by occupational social grade, to provide an indication of changes across the period.

We used binary logistic regression to analyze associations between socioeconomic position and smoking prevalence among adults in England, quit attempts among past-year smokers, use of cessation aids among past-year smokers who made a quit attempt, and quitting success among past-year smokers who made a quit attempt. We used ordinal logistic regression to analyze associations between socioeconomic position and motivation to stop smoking among people who currently smoke, as an equivalent increase in motivation to stop smoking by each one-level increase in the scale score could not be assumed. We used linear regression to analyze associations between socioeconomic position and level of tobacco addiction among past-year smokers, as a more consistent change in level of tobacco addiction between each one-level increase in scale score could be assumed. We tested associations with each indicator of socioeconomic position in separate models, with and without adjustment for covariates. For each measure of socioeconomic position, the reference category was the most advantaged subcategory.

We used complete case data on a per-analysis basis and reported the percentage of missing data for each variable.

## Results

A total of 195 543 adults (≥18 years) in England were surveyed, of whom 36 341 were past-year smokers, 33 113 respondents were current smokers, and 11 739 had tried to quit smoking within the last year. Overall, the mean age of respondents was 49.6 years and 50.1% were women. Further total and subsample demographic smoking and quitting characteristics are listed in Supplementary Table S2.

**Figure 1:**
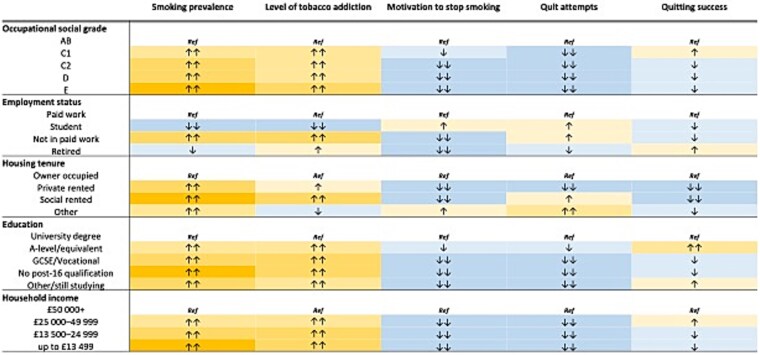
Direction of effect plot for outcomes: smoking prevalence, level of tobacco addiction, motivation to stop smoking, quit attempts, and quitting success by each indicator of socioeconomic position and subcategories. Footnotes: Adjusted, weighted dataset.

**Figure 2:**
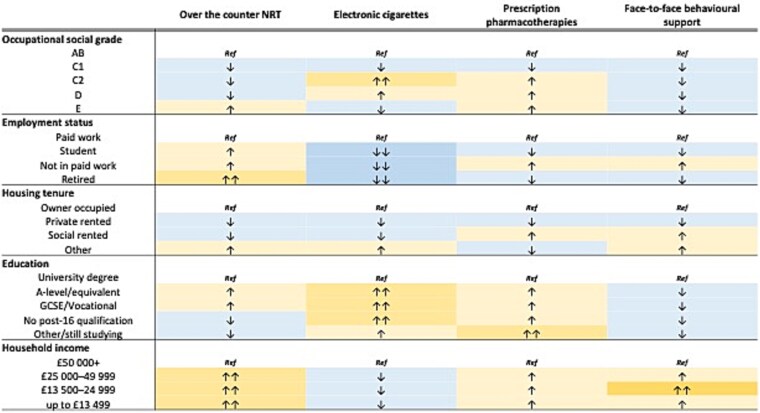
Direction of effect plot for selected cessation aids by each indicator of socioeconomic position and subcategories.

Adjusted associations between each exposure and outcome are reported in text, tables, and summarized graphically in Figures 1 and 2, and in Supplementary Figure S1. Where relevant, unadjusted associations are reported alongside adjusted associations. All analyses on unweighted data are reported in Supplementary Tables (S4, S6, S9, S12, S14, S16, S18, S20, S22, S24, S26, S28, S30, S32, S34).

### Smoking Prevalence

The odds of smoking were higher with increasing disadvantage when measured by occupational social grade, housing tenure, educational level, and household income (Supplementary Table S3). Compared with those in paid work, the odds of smoking were higher among those not in paid work and lower among students.

**Figure 3:**
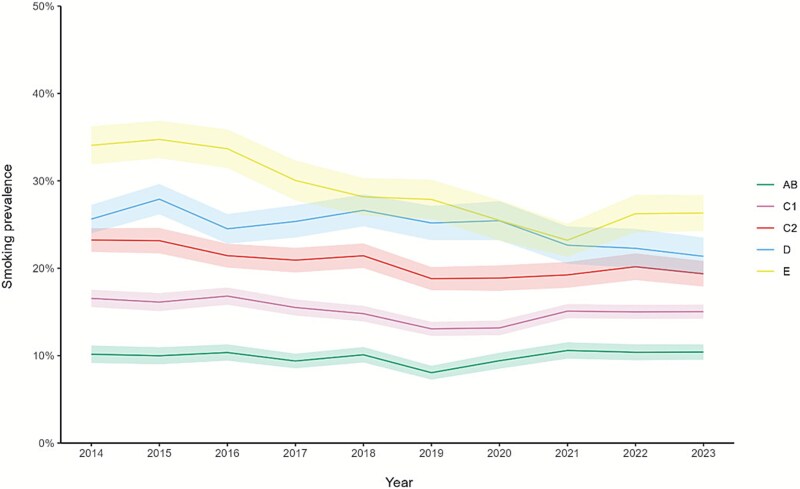
Annual trends in smoking prevalence by occupational social grade over a 10-year period. Footnotes: AB: higher managerial, administrative or professional. C1: supervisory or clerical and junior managerial, administrative or professional. C2: skilled manual workers. D: semi-skilled and unskilled manual workers. E: casual or lowest grade workers, pensioners, and others who depend on the welfare state for their income. Adjusted, weighted dataset.

Figure 3 shows annual trends in smoking prevalence by occupational social grade over a 10-year period. Smoking prevalence generally decreased for all social grades until 2019. Between 2019 and 2021, there was a slight increase in the most advantaged social grades (AB and C1), a plateau in C2, and a continued decline in the most disadvantaged social grades (D and E). Prevalence was then relatively stable between 2021 and 2023 in AB, C1, and C2, declined in D, and increased in E.

### Motivation to Stop Smoking

Among people who currently smoked, those from less advantaged occupational social grades (excluding C1), with lower household income, not in paid work or retired, with less education, or not living in their own home were less motivated to stop smoking compared with people in the most advantaged category for each of these measures of socioeconomic position (Supplementary Table S5). The comparison between those in paid work and students was inconclusive. The percentage of respondents for each motivation to stop smoking response category and socioeconomic position subcategory are reported in Supplementary Table S7.

### Level of Tobacco Addiction

Among past-year smokers, the mean strength of urges to smoke was higher with increasing disadvantage when measured by occupational social grade, educational level, and household income (Supplementary Table S8). Relative to those who owned their own home, respondents living in social housing reported stronger urges to smoke. This direction of effect was also consistent when compared to those living in privately rented housing, but this difference was inconclusive.

Relative to those in paid work, students reported weaker urges to smoke, while those not in paid work reported stronger urges, suggesting a greater level of tobacco addiction. The comparison between those in paid work and retired respondents was inconclusive. The percentage of respondents for each strength of urges to smoke response category and socioeconomic position subcategory are reported in Supplementary Table S10.

### Quit Attempts

Among past-year smokers, those from more disadvantaged socioeconomic positions had lower odds of reporting a quit attempt, as measured by occupational social grade, education level, and household income (Supplementary Table S11).

Those living in privately rented accommodation had slightly lower odds of making a quit attempt relative to homeowners. Conversely, the comparison between homeowners and those living in social housing was inconclusive. Greater odds of making a quit attempt were seen in those from “other” housing tenures when compared to those who owned their own home, however, no further information on this “other” category is available.

No clear evidence of a difference in quit attempts was observed between those in paid employment relative to the other employment status categories.

### Use of Cessation Aids

Among past-year smokers who tried to quit, the most used cessation aids were ECs (33.0%) and NRT without prescription (17.7%); 8.1% used prescription medications (NRT, varenicline, or bupropion), and 2.6% used face-to-face behavioral support (Supplementary Table S2). Findings relating to the following cessation aids are reported in the supplementary materials: prescription NRT, bupropion, varenicline, telephone support, written self-help materials, and alternative treatments (Pages 26–27; Supplementary Tables S21–S32).

#### Over the Counter Nicotine Replacement Therapy

Respondents on household incomes up to £49 999 had higher odds of using OTC NRT relative to those on £50 000 or more. Relative to those in paid work, respondents who were retired had higher odds of using OTC NRT. There was a lack of clear evidence of an effect for all other socioeconomic indicators after adjusting for age, gender, survey year, and strength of urges to smoke (Supplementary Table S13).

#### Electronic Cigarettes

**Figure 4:**
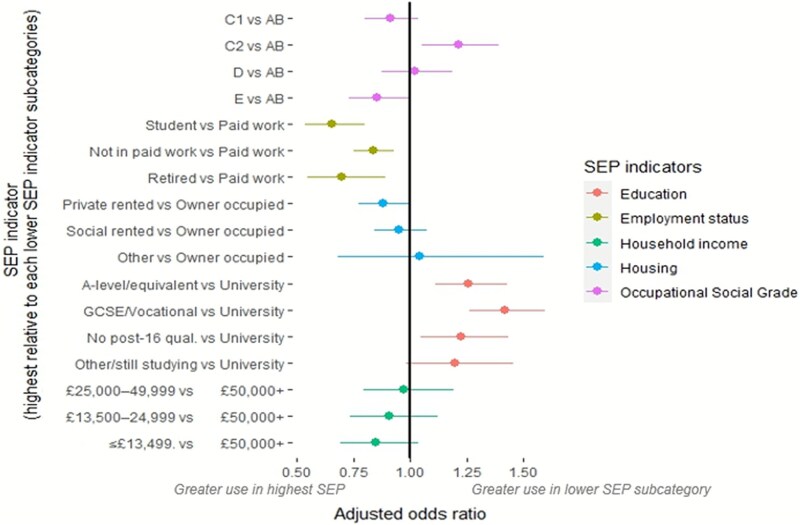
Electronic cigarette cessation aid use relative to the highest subcategory for each SEP indicator. Footnotes: SEP, socioeconomic position; OR, odds ratio; Q, quartile, qual, qualification. vs, versus. Socioeconomic position (ordered highest to lowest socioeconomic subcategories): • ***Occupational social grade:*** AB (higher managerial, administrative or professional); C1 (supervisory or clerical and junior managerial, administrative or professional); C2 (Skilled manual workers); D (Semi-skilled and unskilled manual workers), E (Casual or lowest grade workers, pensioners, and others who depend on the welfare state for their income). • ***Employment status:*** paid work; student; not in paid work; retired. • ***Housing tenure***: owner occupied; private rented (renting from private landlord); social rented (rented from local authority/housing association; other. • ***Educational level:*** University degree, A-level or equivalent (high school senior); General Certificate of Secondary Education (GCSE)/O-level/CSE (high school sophomore) or vocational qualification (high school senior); No post-16 formal qualifications; Other or still studying. • ***Annual household income:*** £50 000+; £25 000–49 999; £13 500–24 999; up to £13 499.Adjusted, weighted dataset.

Use of e-cigarettes in quit attempts varied across the different measures of socioeconomic position (Supplementary Table S15; Figure 4).

Relative to those in the most advantaged occupational social grade, those in the mid-range (C2) had higher odds of using e-cigarettes for smoking cessation, while the direction of effect for those in the lowest social grade (E) favored lower odds, although this comparison was inconclusive.

Relative to those in paid work, respondents in every other employment status category had lower odds of using an e-cigarette as a smoking cessation aid. Conversely, when compared to the highest education level, every other category (excluding other/still studying) had higher odds of using an e-cigarette.

Directions of effect for housing tenure (excluding other) and household income favored greater use of e-cigarettes in the most advantaged groups, however there was a lack of clear evidence of an association.

#### Prescription Medication/s

There was no clear evidence that the use of prescription medications (prescription NRT, varenicline or bupropion) differed by any measure of socioeconomic position. Directions of effects generally suggested greater use of prescribed medication in more disadvantaged respondents, however confidence intervals for all estimates were inconclusive (Supplementary Table S17). Each of these prescription medications are investigated individually, with findings reported in Supplementary Tables S21–S26 and in Supplementary Figure S1.

#### Face-to-Face Behavioral Support

There was no clear evidence that the use of face-to-face behavioral support differed by occupational social grade, employment status, housing tenure or education level after adjusting for age, gender, survey year, and strength of urges to smoke (Supplementary Table S19). Directions of effect varied by socioeconomic indicator and included the possibility of no difference for all except for respondents with an income of £13 500–24 999, who had higher odds of using face-to-face behavioral support compared with those in the highest household income group.

### Quitting Success

Among past-year smokers who tried to quit, respondents who privately rented or were living in social housing had lower odds of quitting successfully compared to those who occupied their own homes, after adjusting for age, gender, survey year, and strength of urges to smoke (Supplementary Table S33).

There was no clear evidence that quitting success differed by other markers of socioeconomic position, although directions of effect suggested lower odds of success in more disadvantaged groups. When looking at education level, those with A-level or equivalent education were slightly more likely to successfully quit compared with those who attended university.

## Discussion

Across all measures of socioeconomic position, increasing disadvantage was associated with higher odds of smoking, higher levels of tobacco addiction, and lower motivation to stop smoking. People from more disadvantaged occupational social grades, on lower household incomes and with less education had lower odds of having made a quit attempt in the past year relative to those in the most advantaged categories. Odds of quitting success were lower in more disadvantaged housing tenures; however no clear evidence was observed when looking at other indicators of socioeconomic position.

E-cigarettes were the most used smoking cessation aid, and odds of use by socioeconomic position varied greatly by indicator. Students and people who were retired or not in paid work were less likely to use e-cigarettes compared to those in paid employment, while people with less education were more likely to use e-cigarettes. Disadvantage was associated with greater use of prescription NRT, and less use of varenicline and written self-help materials, when compared to the most advantaged, however associations did not meet statistical significance thresholds for all indicators. Respondents on lower household incomes, or who were retired as opposed to in paid employment, had higher odds of using over the counter NRT, however evidence across other indicators were inconclusive. Evidence on use of all other cessation aids by socioeconomic position varied across different indicators or were inconclusive with varying directions of effect.

### Similarities and Differences across Indicators of Socioeconomic Position

Findings were largely consistent across different indicators of socioeconomic position for all outcomes, except use of cessation aids for which it either varied greatly or for which we had no clear evidence. Differences found across cessation aid use and types of socioeconomic indicators may be related to other factors, such as cost (e.g. lower odds of e-cigarette use if not in paid employment), perceptions of the aid, or access to specific cessation aids.

A previous analysis of the same survey found that people living in social compared to other housing were more likely to use e-cigarettes and equally motivated to quit smoking,^13^ which appears to contradict the direction of effect and findings for housing tenure found in this study. This can be explained by the use of different comparator groups: here, we analyzed each housing type as a separate category, whereas the previous study compared social housing with all other housing types grouped together. This highlights that associations may vary by the level of stratification of deprivation explored. Further explanations for these discrepancies may be due to the binary categorization of the motivation to quit variable (compared to ordinal categorization used in this study), the smaller sample and/or the adjustment for additional socioeconomic covariates (occupational social grade and government office region) used in this other study.

Disadvantage was associated with lower odds of making a quit attempt for all socioeconomic indicators except for employment status and social housing for which findings indicated opposing directions of effect, however lacked clear evidence. Associations between quitting success and most socioeconomic indicators were also inconclusive. Previous research has highlighted that people from less advantaged socioeconomic groups are less likely to succeed in achieving abstinence compared with those from more advantaged groups.^9^^,^^13^^,^^20^ Our findings when focused on housing tenure showed a similar pattern, however no clear evidence was observed when looking at all other indicators of socioeconomic position. Some evidence has identified housing tenure as the strongest independent predictor of smoking status in England,^21^ which may warrant a greater consideration being given to housing tenure when investigating associations between quitting success and socioeconomic position in this context.

### Implications for Research and Practice

Findings suggest that further research investigating smoking prevalence, level of tobacco addiction, motivation to stop smoking, quit attempts, and quitting success may not need to assess multiple indicators of socioeconomic position when exploring relationships between these outcomes and disadvantaged groups, as results tend to be fairly similar regardless of the measure used to assess socioeconomic position. However, this does not necessarily imply that tailoring smoking cessation interventions for people with, for example, less education or on less income, will equivalently address socioeconomic inequalities in smoking, or that one type of tailored intervention will be similar in effectiveness across different forms, or degree, of disadvantage.

### Strengths and Limitations

Our use of a large, representative dataset allowed for detailed exploration of varying types and levels of socioeconomic position, as measured by multiple indicators in a real-world setting. However, the nature of this design imposed inherent limitations common to observational studies. Firstly, data were self-reported and some outcomes relied on recall of the previous year. However, while this may introduce some bias, we would not expect this to differ substantially by socioeconomic position. Secondly, while we adjusted for many relevant covariates, other factors associated with smoking and quitting success, such as social norms and self-efficacy were not accounted for.^22–24^ Furthermore, no further information on some categories such as “other” and “other/still studying” (for housing tenure and education level, respectively) were collected as part of the STS survey, which limited interpretation of findings for these categories. Due to the lack of longitudinal follow-up, we cannot draw inferences regarding cause and effect.

Sample sizes for some cessation aids were relatively small which led to high uncertainty in some of the estimated effects. As such, we focused discussion on the more frequently used cessation aids. There was also some missing data, and for transparency we reported the percentage missing by each variable. We assessed e-cigarette use as a cessation aid; however, level of tobacco addiction may have been impacted by recreational use in dual e-cigarette and combustible cigarette users. Finally, it should be noted that the sample analyzed was from England only, which may limit generalizability of findings to other countries or regions with different tobacco regulations and social contexts.

## Conclusions

In England, there was consistent evidence that people experiencing greater socioeconomic disadvantage were substantially more likely to smoke than those who were more advantaged, had higher levels of tobacco addiction, and were less motivated to stop smoking. These findings were consistent when measured by different indicators of socioeconomic position, including occupational social grade, employment, housing tenure, education and household income, which suggests use of any one of these indicators is likely to show consistent findings when investigating relationships between socioeconomic position and smoking prevalence, tobacco addiction, and motivation to quit. Evidence on quitting was less consistent: according to some but not all socioeconomic indicators, disadvantage was associated with lower odds of making a quit attempt. Odds of quitting success were lower in more disadvantaged housing tenures; however, there was no clear evidence when using other indicators of socioeconomic position. There were some differences in use of e-cigarettes as a cessation aid by socioeconomic position, but patterning of this was not yet clear for all socioeconomic indicators. Evidence on the use of other cessation aids was limited by small samples or uncertainty.

## Supplementary Material

Supplementary_material_Final_16_Oct_24_V4_23_Sep_2025_ntag003

## Data Availability

The datasets used and/or analyzed during the current study are available from the senior author on reasonable request.

## Notes

Protocol registration details: The protocol was registered *a priori* on: https://osf.io/kgaz9/
