# Supplementary material for: Smoking and Quitting Behaviors by Different Indicators of Socioeconomic Position in England: A Population Study, 2014 to 2023
Source: Nicotine Tob Res. 2026 Feb 11;28(6):1045–52. doi: 10.1093/ntr/ntag003 (PMC13196705; doi:10.1093/ntr/ntag003)
Supplement: Supplementary_material_Final_16_Oct_24_V4_23_Sep_2025_ntag003 [file supplementary_material_final_16_oct_24_v4_23_sep_2025_ntag003.docx]

Supplementary material

**Smoking and quitting behaviours by different indicators of socioeconomic position in England: a population study, 2014 to 2023**

**Authors**

Annika Theodoulou^1^, Jamie Hartmann-Boyce^2^, Nicola Lindson^1^, Thomas R Fanshawe^1^, Sarah E Jackson^3^

1. Nuffield Department of Primary Care Health Sciences, University of Oxford, United Kingdom
2. Department of Health Promotion and Policy, University of Massachusetts, Amherst, MA, United States of America
3. Department of Behavioural Science and Health, University College London, London, United Kingdom

Table of Contents

Research questions and demographics characteristics 5

Table S1: Research question, outcome and respondent data category 5

Table S2: Demographic, smoking and quitting characteristics for all respondents and respondent subgroups (current smokers, past-year smokers and past-year smokers who made a quit attempt) 6

Smoking prevalence by socioeconomic position 10

Research question 1 10

Table S3: Smoking prevalence by socioeconomic position (weighted dataset) 11

Table S4: Smoking prevalence by socioeconomic position (unweighted dataset) 12

Motivation to stop smoking by socioeconomic position 13

Research question 2 13

Table S5: Motivation to stop smoking by socioeconomic position among current smokers (weighted dataset) 14

Table S6: Motivation to stop smoking by socioeconomic position (unweighted dataset) 15

Table S7: Percentage by motivation to stop smoking response categories by each SES subcategories 16

Level of tobacco addiction by socioeconomic position 18

Research question 3 18

Table S8: Level of tobacco addiction (strength of urges to smoke) by socioeconomic position among past-year smokers (weighted dataset) 19

Table S9: Level of tobacco addiction (strength of urges to smoke) by socioeconomic position (unweighted dataset) 20

Table S10: Level of tobacco addiction (strength of urges to smoke) by each SES subcategories 21

Quit attempts by socioeconomic position 23

Research question 4 23

Table S11: Quit attempts by socioeconomic position among past-year smokers (weighted dataset) 24

Table S12: Quit attempts by socioeconomic position in past-year smokers (unweighted dataset) 25

Use of cessation aids by socioeconomic position 26

Research question 5 26

Results for a selection of cessation aids 26

Prescription nicotine replacement therapy 26

Bupropion 26

Varenicline 26

Telephone support 26

Written self-help materials 27

Alternative treatments 27

Table S13: Use of over the counter NRT by socioeconomic position (weighted dataset) 28

Table S14: Use of over the counter NRT by socioeconomic position (unweighted dataset) 29

Table S15: Use of electronic cigarettes by socioeconomic position (weighted dataset) 30

Table S16: Use of electronic cigarettes by socioeconomic position (unweighted dataset) 31

Table S17: Use of prescription pharmacotherapies (NRT with prescription, Bupropion and Varenicline) by socioeconomic position (weighted dataset) 32

Table S18: Use of prescription pharmacotherapies (NRT with prescription, Bupropion and Varenicline) by socioeconomic position (unweighted dataset) 33

Table S19: Use of face-to-face behavioural support by socioeconomic position (weighted dataset) 34

Table S20: Use of face-to-face behavioural support by socioeconomic position (unweighted dataset) 35

Table S21: Use of prescription NRT by socioeconomic position (weighted dataset) 36

Table S22: Use of prescription NRT by socioeconomic position (unweighted dataset) 37

Table S23: Use of bupropion by socioeconomic position (weighted dataset) 38

Table S24: Use of bupropion by socioeconomic position (unweighted dataset) 39

Table S25: Use of varenicline by socioeconomic position (weighted dataset) 40

Table S26: Use of varenicline by socioeconomic position (unweighted dataset) 41

Table S27: Use of telephone support by socioeconomic position (weighted dataset) 42

Table S28: Use of telephone support by socioeconomic position (unweighted dataset) 43

Table S29: Use of written self-help materials by socioeconomic position (weighted dataset) 44

Table S30: Use of written self-help materials by socioeconomic position (unweighted dataset) 45

Table S31: Use of alternative treatments by socioeconomic position (weighted dataset) 46

Table S32: Use of alternative treatments by socioeconomic position (unweighted dataset) 47

Figure S1: Direction of effect plot for cessation aids by each indicator of socioeconomic position and subcategories 48

Quitting success 50

Research question 5 50

Table S33: Quitting success by socioeconomic position among past-year smokers who made a quit attempt 51

Table S34: Quitting success by socioeconomic position (unweighted dataset) 52

Protocol deviations 53

# Research questions and demographics characteristics

## Table S1: Research question, outcome and respondent data category

| ***Research question (RQ)*** | ***Outcome(s)*** | ***Respondent category*** |
| --- | --- | --- |
| RQ1 | Smoking prevalence^a^ | All respondents |
| RQ2 | Motivation to stop smoking | Current smoker^a^ (responses 1-3) |
| RQ3 | Level of cigarette addiction  (measured by strength of urges to smoke) | Past-year smoker^a^ (responses 1-4) |
| RQ4 | Past-year quit attempts | Past-year smoker^a^ (responses 1-4) |
| RQ5 | Use of cessation aids  Quitting success | Past-year smoker^a^ (responses 1-4) who made a quit attempt |
| ^a^ Respondent categories will be based on participant responses to the question *“Which of the following best applies to you?”*   1. I smoke cigarettes (including handrolled) every day 2. I smoke cigarettes (including hand-rolled), but not every day 3. I do not smoke cigarettes at all, but I do smoke tobacco of some kind (e.g. Pipe, cigar or shisha) 4. I have stopped smoking completely in the last year 5. I stopped smoking completely more than a year ago 6. I have never been a smoker (i.e. smoked for a year or more)   Smoking prevalence was categorised as ‘currently smoking’ (responses 1 to 3) or ‘not currently smoking’ (responses 4 to 6) | | |

## Table S2: Demographic, smoking and quitting characteristics for all respondents and respondent subgroups (current smokers, past-year smokers and past-year smokers who made a quit attempt)

|  | **All respondents**  *(N = 195, 543)* | | | | **Current smokers**  *(N = 33, 113)* | | | | **Past-year smokers**  *(N = 36, 341)* | | | | **Past-year smokers who made a quit attempt**  *(N =11, 739)* | | | |
| --- | --- | --- | --- | --- | --- | --- | --- | --- | --- | --- | --- | --- | --- | --- | --- | --- |
|  | ***N^a^*** | | ***%^b^*** | | ***N^a^*** | | ***% ^b^*** | | ***N^a^*** | | ***% ^b^*** | | ***N^a^*** | | ***% ^b^*** | |
| Age (years) |  |  |  |  |  |  |  |  |  |  |  |  |  |  |  |  |
| 18 – 24 | 24732 | | 12.4 | | 5690 | | 16.6 | | 6307 | | 16.8 | | 2340 | | 19.2 | |
| 24 – 34 | 29188 | | 17.2 | | 6966 | | 23.8 | | 7790 | | 24.2 | | 2921 | | 27.6 | |
| 35 – 44 | 28167 | | 16.3 | | 5373 | | 18 | | 5941 | | 18.1 | | 2048 | | 19 | |
| 45 – 54 | 31007 | | 17.2 | | 5563 | | 17.5 | | 6049 | | 17.3 | | 1831 | | 15.8 | |
| 55 – 64 | 31483 | | 14.6 | | 4790 | | 12.6 | | 5177 | | 12.3 | | 1454 | | 10.6 | |
| 65+ | 50966 | | 22.3 | | 4731 | | 11.6 | | 5077 | | 11.3 | | 1145 | | 7.85 | |
| Gender |  |  |  |  |  |  |  |  |  |  |  |  |  |  |  |  |
| Male | 97247 | | 49.0 | | 17565 | | 53.5 | | 19231 | | 53.5 | | 5968 | | 51.9 | |
| Female | 97581 | | 51.0 | | 15358 | | 46.5 | | 16899 | | 46.5 | | 5698 | | 48.1 | |
| Missing | 715 | | - | | 190 | |  | | 211 | | - | | 73 | | - | |
| Occupational social grade |  |  |  |  |  |  |  |  |  |  |  |  |  |  |  |  |
| AB *_highest_* | 47708 | | 27.1 | | 4541 | | 15.3 | | 5128 | | 15.7 | | 1738 | | 16.7 | |
| C1 | 70542 | | 28.4 | | 10437 | | 24.5 | | 11676 | | 24.9 | | 3943 | | 25.9 | |
| C2 | 35618 | | 21.1 | | 7184 | | 24.9 | | 7828 | | 24.8 | | 2456 | | 24.1 | |
| D | 22616 | | 14.6 | | 5528 | | 20.6 | | 5936 | | 20.3 | | 1835 | | 19.7 | |
| E _Lowest_ | 19059 | | 8.9 | | 5423 | | 14.7 | | 5773 | | 14.2 | | 1767 | | 13.6 | |
| Employment status |  | |  | |  | |  | |  | |  | |  | |  | |
| Paid work | 103410 | | 58.0 | | 18122 | | 60.0 | | 20149 | | 60.6 | | 6792 | | 62.4 | |
| Student | 11749 | | 5.4 | | 1813 | | 4.7 | | 2029 | | 4.8 | | 749 | | 5.48 | |
| Not in paid work | 25731 | | 12.9 | | 7781 | | 22.2 | | 8346 | | 21.7 | | 2832 | | 22.8 | |
| Retired | 54341 | | 23.8 | | 5333 | | 13.1 | | 5749 | | 12.8 | | 1349 | | 9.32 | |
| Missing | 312 | | - | | 64 | | - | | 68 | | - | | 17 | | - | |
| Housing tenure |  | |  | |  | |  | |  | |  | |  | |  | |
| Owner occupied | 87895 | | 64.6 | | 9833 | | 42.4 | | 10769 | | 43.0 | | 3213 | | 41.4 | |
| Private rented | 27953 | | 20.7 | | 7107 | | 30.8 | | 7686 | | 30.8 | | 2437 | | 30.7 | |
| Social rented | 23178 | | 13.6 | | 7513 | | 25.6 | | 7947 | | 25.1 | | 2578 | | 26.3 | |
| Other | 1456 | | 1.1 | | 260 | | 1.2 | | 292 | | 1.2 | | 117 | | 1.6 | |
| Missing | 55061 | | - | | 8400 | | - | | 9647 | | - | | 3394 | | - | |
| Education level |  | |  | |  | |  | |  | |  | |  | |  | |
| University education | 67802 | | 35.6 | | 7180 | | 21.9 | | 8218 | | 22.9 | | 2931 | | 25.2 | |
| A-level or equivalent | 37657 | | 19.6 | | 6966 | | 21.5 | | 7749 | | 21.7 | | 2771 | | 23.8 | |
| GCSE/Vocational | 47017 | | 24.8 | | 10585 | | 33.4 | | 11425 | | 32.8 | | 3689 | | 32.5 | |
| No post-16 qualification | 27236 | | 13.0 | | 5725 | | 16.2 | | 6056 | | 15.6 | | 1528 | | 12.3 | |
| Other/still studying ^c^ | 14346 | | 7.0 | | 2350 | | 7.0 | | 2566 | | 7.0 | | 736 | | 6.3 | |
| Missing | 1485 | | - | | 307 | | - | | 327 | | - | | 84 | | - | |
| Annual household income |  | |  | |  | |  | |  | |  | |  | |  | |
| £50 000+ | 16747 | | 25 | | 1803 | | 14.7 | | 2037 | | 15.3 | | 740 | | 16.8 | |
| £25 000–49 999 | 23695 | | 32.2 | | 3906 | | 29.6 | | 4261 | | 29.9 | | 1414 | | 30.0 | |
| £13 500–24 999 | 16056 | | 20.1 | | 3342 | | 23.8 | | 3577 | | 23.5 | | 1166 | | 23.1 | |
| up to £13 499 | 19453 | | 22.6 | | 5013 | | 32.0 | | 5318 | | 31.3 | | 1674 | | 30.1 | |
| Missing | 119592 | | - | | 19049 | | - | | 21148 | | - | | 6745 | | - | |
| Motivation to stop smoking |  | |  | |  | |  | |  | |  | |  | |  | |
| 1 – lowest: do not want to stop | - | | - | | 9770 | | 29.3 | | - | | - | | - | | - | |
| 2 | - | | - | | 5439 | | 16.8 | | - | | - | | - | | - | |
| 3 | - | | - | | 3238 | | 10.2 | | - | | - | | - | | - | |
| 4 | - | | - | | 4836 | | 14.9 | | - | | - | | - | | - | |
| 5 | - | | - | | 4390 | | 13.4 | | - | | - | | - | | - | |
| 6 | - | | - | | 2600 | | 8.1 | | - | | - | | - | | - | |
| 7 – highest: Really want to stop and intend to in <1 month | - | | - | | 2383 | | 7.3 | | - | | - | | - | | - | |
| Missing | - | | - | | 3685 | | - | | - | | - | | - | | - | |
| Level of tobacco addiction  (strength of urges to smoke) |  | |  | |  | |  | |  | |  | |  | |  | |
| Not at all *_lowest_* | - | | - | | - | | - | | 6954 | | 19.7 | | - | | - | |
| Slight | - | | - | | - | | - | | 6238 | | 17.8 | | - | | - | |
| Moderate | - | | - | | - | | - | | 14451 | | 40.7 | | - | | - | |
| Strong | - | | - | | - | | - | | 5493 | | 15.0 | | - | | - | |
| Very strong | - | | - | | - | | - | | 1689 | | 4.7 | | - | | - | |
| Extremely strong *_Highest_* | - | | - | | - | | - | | 812 | | 2.2 | | - | | - | |
| Missing | - | | - | | - | | - | | 704 | | - | | - | | - | |
| Quit attempts in the last year |  | |  | |  | |  | |  | |  | |  | |  | |
| No serious quit attempt |  | |  | |  | |  | | 23214 | | 65.9 | |  | |  | |
| Made ≥1 serious quit attempt | - | | - | | - | | - | | 11739 | | 34.1 | |  | |  | |
| Missing | - | | - | | - | | - | | 1388 | | - | | - | | - | |
| Use of different cessation aids^d^ |  | |  | |  | |  | |  | |  | |  | |  | |
| Over the counter NRT | - | | - | | - | | - | | - | | - | | 2116 | | 17.7 | |
| Prescription NRT | - | | - | | - | | - | | - | | - | | 479 | | 4.0 | |
| Electronic cigarettes | - | | - | | - | | - | | - | | - | | 3774 | | 33.0 | |
| Combined prescription medications^e^ | - | | - | | - | | - | | - | | - | | 952 | | 8.1 | |
| Bupropion | - | | - | | - | | - | | - | | - | | 72 | | 0.6 | |
| Varenicline | - | | - | | - | | - | | - | | - | | 462 | | 4.1 | |
| Face-to-face behavioural support | - | | - | | - | | - | | - | | - | | 308 | | 2.6 | |
| Telephone support | - | | - | | - | | - | | - | | - | | 76 | | 0.7 | |
| Written self-help materials | - | | - | | - | | - | | - | | - | | 590 | | 5.2 | |
| Alternative treatments | - | | - | | - | | - | | - | | - | | 119 | | 1.1 | |
|  |  | |  | |  | |  | |  | |  | |  | |  | |
| *NRT: nicotine replacement therapy*  *^a^ Number of participants (unweighted dataset);*  *^b^ Percentages based on weighted dataset excluding missing data;*  *^c^ combined ‘Other’ and ‘still studying’ response options: Number of respondents for ‘Other’ = 12,662 (88.3%); ‘Still studying’ = 1,684 (11.7%)*  *^d^ See protocol table 7 for further information on cessation aid descriptions*  *^e^ combination prescription medication including prescription NRT, bupropion and varenicline*  *- not applicable* | | | | | | | | | | | | | | | | |

# Smoking prevalence by socioeconomic position

## Research question 1

Among adults in England, to what extent does smoking prevalence differ by socioeconomic position after adjusting for age, gender, and survey year?

## Table S3: Smoking prevalence by socioeconomic position (weighted dataset)

| **SEP indicator** | **N^a^** | **Smoking prevalence, %^b^** | **OR [95%CI]^b^** | **OR_adj_ [95%CI]^b^** |  |
| --- | --- | --- | --- | --- | --- |
| ***Occupational social grade*** |  |  |  |  |  |
| AB | 47578 | 9.9 | *ref* | *ref* |  |
| C1 | 70303 | 15.1 | 1.62 [1.56, 1.69] | 1.55 [1.49, 1.61] |  |
| C2 | 35523 | 20.7 | 2.39 [2.29, 2.49] | 2.31 [2.21, 2.41] |  |
| D | 22543 | 24.7 | 3.00 [2.86, 3.14] | 2.91 [2.78, 3.05] |  |
| E | 18994 | 28.9 | 3.71 [3.54, 3.89] | 4.36 [4.16, 4.58] |  |
|  |  |  |  |  |  |
| ***Employment status*** |  |  |  |  |  |
| Paid work | 103048 | 18.1 | *ref* | *ref* |  |
| Student | 11729 | 15.4 | 0.82 [0.77, 0.87] | 0.57 [0.53, 0.61] |  |
| Not in paid work | 25652 | 30.1 | 1.95 [1.88, 2.01] | 2.04 [1.97, 2.11] |  |
| Retired | 54217 | 9.6 | 0.48 [0.46, 0.50] | 0.94 [0.88, 1.00] |  |
| Missing | 295 | 22.8 | - | - |  |
|  |  |  |  |  |  |
| ***Housing tenure*** |  |  |  |  |  |
| Owner occupied | 87811 | 11.7 | *ref* | *ref* |  |
| Private rented | 27921 | 26.5 | 2.72 [2.62, 2.82] | 2.28 [2.18, 2.37] |  |
| Social rented | 23132 | 33.6 | 3.82 [3.68, 3.96] | 3.70 [3.56, 3.84] |  |
| Other | 1452 | 18.4 | 1.70 [1.47, 1.97] | 1.54 [1.32, 1.78] |  |
| Missing | 54625 | 16.6 | - | - |  |
|  |  |  |  |  |  |
| ***Education*** |  |  |  |  |  |
| University degree | 67561 | 10.8 | *ref* | *ref* |  |
| A-level/equivalent | 37552 | 19.1 | 1.96 [1.89, 2.04] | 1.88 [1.80, 1.96] |  |
| GCSE/Vocational | 46901 | 23.6 | 2.55 [2.46, 2.65] | 2.85 [2.75, 2.96] |  |
| No post-16 qualification | 27151 | 21.7 | 2.30 [2.21, 2.40] | 3.85 [3.68, 4.03] |  |
| Other/still studying | 14302 | 17.4 | 1.74 [1.65, 1.84] | 2.11 [1.99, 2.23] |  |
| Missing | 1474 | 22.1 | - | - |  |
|  |  |  |  |  |  |
| ***Household income*** |  |  |  |  |  |
| £50 000+ | 16742 | 10.9 | *ref* | *ref* |  |
| £25 000–49 999 | 23682 | 17.0 | 1.68 [1.58, 1.79] | 1.82 [1.70, 1.94] |  |
| £13 500–24 999 | 16048 | 22.0 | 2.31 [2.16, 2.47] | 2.83 [2.64, 3.03] |  |
| up to £13 499 | 19444 | 26.2 | 2.91 [2.73, 3.10] | 3.78 [3.54, 4.04] |  |
| Missing | 119025 | 16.8 | - | - |  |
| CI, confidence interval. OR, odds ratio. OR_adj_, odds ratio adjusted for age, gender, and survey year. SEP, socioeconomic position.  ^a^ Unweighted sample size.  ^b^ weighted dataset (refer to supplementary Table S4 for unweighted dataset percentages and effect estimates.  - not applicable | | | | | |

## Table S4: Smoking prevalence by socioeconomic position (unweighted dataset)

| **SEP indicator** | **N** | **Smoking prevalence, %** | **OR [95%CI]** | **OR_adj_ [95%CI]** |  |
| --- | --- | --- | --- | --- | --- |
| ***Occupational social grade*** |  |  |  |  |  |
| AB | 47578 | 9.5 | *ref* | *ref* |  |
| C1 | 70303 | 14.8 | 1.62 [1.56, 1.69] | 1.55 [1.50, 1.61] |  |
| C2 | 35523 | 20.2 | 2.39 [2.29, 2.49] | 2.31 [2.22, 2.41] |  |
| D | 22543 | 24.5 | 3.00 [2.86, 3.14] | 2.89 [2.77, 3.02] |  |
| E | 18994 | 28.6 | 3.71 [3.54, 3.89] | 4.29 [4.10, 4.49] |  |
|  |  |  |  |  |  |
| ***Employment status*** |  |  |  |  |  |
| Paid work | 103048 | 17.6 | *ref* | *ref* |  |
| Student | 11729 | 15.5 | 0.86 [0.81, 0.90] | 0.57 [0.54, 0.61] |  |
| Not in paid work | 25652 | 30.3 | 2.04 [1.98, 2.10] | 2.08 [2.02, 2.15] |  |
| Retired | 54217 | 9.8 | 0.51 [0.49, 0.53] | 0.95 [0.90, 1.01] |  |
| Missing | 295 | 21.7 | - | - |  |
|  |  |  |  |  |  |
| ***Housing tenure*** |  |  |  |  |  |
| Owner occupied | 87811 | 11.2 | *ref* | *ref* |  |
| Private rented | 27921 | 25.5 | 2.71 [2.62, 2.80] | 2.23 [2.15, 2.32] |  |
| Social rented | 23132 | 32.5 | 3.81 [3.68, 3.95] | 3.66 [3.53, 3.79] |  |
| Other | 1452 | 17.9 | 1.73 [1.51, 1.98] | 1.55 [1.35, 1.78] |  |
| Missing | 54625 | 15.4 | - | - |  |
|  |  |  |  |  |  |
| ***Education*** |  |  |  |  |  |
| University degree | 67561 | 10.6 | *ref* | *ref* |  |
| A-level/equivalent | 37552 | 18.6 | 1.92 [1.85, 1.98] | 1.82 [1.76, 1.89] |  |
| GCSE/Vocational | 46901 | 22.6 | 2.45 [2.37, 2.53] | 2.74 [2.65, 2.84] |  |
| No post-16 qualification | 27151 | 21.1 | 2.25 [2.16, 2.33] | 3.71 [3.56, 3.87] |  |
| Other/still studying | 14302 | 16.4 | 1.65 [1.57, 1.74] | 2.01 [1.91, 2.12] |  |
| Missing | 1474 | 20.8 | - | - |  |
|  |  |  |  |  |  |
| ***Household income*** |  |  |  |  |  |
| £50 000+ | 16742 | 10.8 | *ref* | *ref* |  |
| £25 000–49 999 | 23682 | 16.5 | 1.64 [1.54, 1.74] | 1.81 [1.70, 1.92] |  |
| £13 500–24 999 | 16048 | 20.8 | 2.18 [2.05, 2.32] | 2.72 [2.55, 2.90] |  |
| up to £13 499 | 19444 | 25.8 | 2.88 [2.72, 3.05] | 3.72 [3.50, 3.96] |  |
| Missing | 119025 | 16.0 | - | - |  |
| CI, confidence interval. OR, odds ratio. OR_adj_, odds ratio adjusted for age, gender, and survey year. SEP, socioeconomic position.  - not applicable | | | | | |

# Motivation to stop smoking by socioeconomic position

## Research question 2

Among people who currently smoke, to what extent does motivation to stop smoking differ by socioeconomic position after adjusting for age, gender, and survey year?

## Table S5: Motivation to stop smoking by socioeconomic position among current smokers (weighted dataset)

| **SEP indicator** | **N** ^a, b^ | **OR [95%CI]^c^** | **OR_adj_ [95%CI]^c^** |
| --- | --- | --- | --- |
| ***Occupational social grade*** |  |  |  |
| AB | 4442 | *ref* | *ref* |
| C1 | 10238 | 0.97 [0.91, 1.04] | 0.96 [0.90, 1.03] |
| C2 | 7113 | 0.85 [0.79, 0.91] | 0.84 [0.79, 0.90] |
| D | 5500 | 0.86 [0.80, 0.92] | 0.84 [0.78, 0.90] |
| E | 5363 | 0.81 [0.75, 0.87] | 0.82 [0.75, 0.88] |
|  |  |  |  |
| ***Employment status*** |  |  |  |
| Paid work | 17847 | *ref* | *ref* |
| Student | 1784 | 1.01 [0.93, 1.10] | 1.09 [1.00, 1.20] |
| Not in paid work | 7707 | 0.95 [0.91, 1.01] | 0.94 [0.89, 0.99] |
| Retired | 5259 | 0.57 [0.54, 0.61] | 0.84 [0.76, 0.94] |
| Missing | 59 | - | - |
|  |  |  |  |
| ***Housing tenure*** |  |  |  |
| Owner occupied | 9761 | *ref* | *ref* |
| Private rented | 7071 | 0.98 [0.93, 1.04] | 0.87 [0.81, 0.92] |
| Social rented | 7486 | 0.98 [0.93, 1.04] | 0.91 [0.86, 0.97] |
| Other | 256 | 1.09 [0.86, 1.37] | 1.04 [0.82, 1.32] |
| Missing | 8082 | - | - |
|  |  |  |  |
| ***Education*** |  |  |  |
| University degree | 7016 | *ref* | *ref* |
| A-level/equivalent | 6867 | 0.94 [0.88, 1.00] | 0.95 [0.89, 1.01] |
| GCSE/Vocational | 10487 | 0.84 [0.80, 0.89] | 0.86 [0.81, 0.91] |
| No post-16 qualification | 5670 | 0.56 [0.52, 0.60] | 0.64 [0.59, 0.69] |
| Other/still studying | 2317 | 0.76 [0.69, 0.84] | 0.82 [0.74, 0.90] |
| Missing | 299 | - | - |
|  |  |  |  |
| ***Household income*** |  |  |  |
| £50 000+ | 1796 | *ref* | *ref* |
| £25 000–49 999 | 3896 | 0.83 [0.75, 0.92] | 0.85 [0.76, 0.94] |
| £13 500–24 999 | 3335 | 0.82 [0.74, 0.91] | 0.85 [0.76, 0.94] |
| up to £13 499 | 5004 | 0.73 [0.66, 0.80] | 0.76 [0.69, 0.85] |
| Missing | 18625 | - | - |
| CI, confidence interval. OR, odds ratio. OR_adj_, odds ratio adjusted for age, gender, and survey year. SEP, socioeconomic position.  ^a^ Unweighted sample size.  ^b^ Percentages for each motivation to stop smoking response category by each SES indicator (weighted dataset) are presented in supplementary table S7.  ^c^ weighted dataset (refer to supplementary Table S6 for unweighted dataset effect estimates). | | | |

## Table S6: Motivation to stop smoking by socioeconomic position (unweighted dataset)

| **SEP indicator** | **N** | **OR [95%CI]** | **OR_adj_ [95%CI]** |
| --- | --- | --- | --- |
| ***Occupational social grade*** |  |  |  |
| AB | 4442 | *ref* | *ref* |
| C1 | 10238 | 0.99 [0.93, 1.05] | 0.97 [0.91, 1.03] |
| C2 | 7113 | 0.85 [0.80, 0.91] | 0.84 [0.79, 0.90] |
| D | 5500 | 0.85 [0.79, 0.91] | 0.83 [0.77, 0.89] |
| E | 5363 | 0.82 [0.76, 0.88] | 0.82 [0.76, 0.88] |
|  |  |  |  |
| ***Employment status*** |  |  |  |
| Paid work | 17847 | *ref* | *ref* |
| Student | 1784 | 1.01 [0.93, 1.10] | 1.10 [1.00, 1.20] |
| Not in paid work | 7707 | 0.93 [0.89, 0.98] | 0.93 [0.88, 0.97] |
| Retired | 5259 | 0.56 [0.53, 0.59] | 0.83 [0.76, 0.92] |
| Missing | 59 | *-* | *-* |
|  |  |  |  |
| ***Housing tenure*** |  |  |  |
| Owner occupied | 9761 | *ref* | *ref* |
| Private rented | 7071 | 1.04 [0.99, 1.10] | 0.89 [0.84, 0.94] |
| Social rented | 7486 | 1.00 [0.95, 1.05] | 0.92 [0.87, 0.97] |
| Other | 256 | 1.16 [0.93, 1.44] | 1.09 [0.87, 1.35] |
| Missing | 8082 | - | - |
|  |  |  |  |
| ***Education*** |  |  |  |
| University degree | 7016 | *ref* | *ref* |
| A-level/equivalent | 6867 | 0.92 [0.87, 0.98] | 0.93 [0.88, 0.99] |
| GCSE/Vocational | 10487 | 0.83 [0.78, 0.87] | 0.84 [0.80, 0.89] |
| No post-16 qualification | 5670 | 0.55 [0.52, 0.59] | 0.64 [0.60, 0.68] |
| Other/still studying | 2317 | 0.74 [0.68, 0.81] | 0.81 [0.74, 0.88] |
| Missing | 299 | *-* | *-* |
|  |  |  |  |
| ***Household income*** |  |  |  |
| £50 000+ | 1796 | *ref* | *ref* |
| £25 000–49 999 | 3896 | 0.84 [0.77, 0.93] | 0.86 [0.78, 0.95] |
| £13 500–24 999 | 3335 | 0.83 [0.75, 0.92] | 0.86 [0.78, 0.95] |
| up to £13 499 | 5004 | 0.74 [0.67, 0.81] | 0.79 [0.72, 0.87] |
| Missing | 18625 | *-* | *-* |
| CI, confidence interval. OR, odds ratio. OR_adj_, odds ratio adjusted for age, gender, and survey year. SEP, socioeconomic position.  - not applicable | | | |

## Table S7: Percentage by motivation to stop smoking response categories by each SES subcategories

|  | **Percentage by motivation to stop smoking categories**  *Lowest (1) to highest (7)* | | | | | | | | | | | | | |
| --- | --- | --- | --- | --- | --- | --- | --- | --- | --- | --- | --- | --- | --- | --- |
| **SEP indicator** | **Weighted dataset** | | | | | | | **Unweighted dataset** | | | | | | |
|  | **1** | **2** | **3** | **4** | **5** | **6** | **7** | **1** | **2** | **3** | **4** | **5** | **6** | **7** |
| **Occupational social grade** |  |  |  |  |  |  |  |  |  |  |  |  |  |  |
| AB *_highest_* | 25.9 | 19.3 | 9.6 | 14.9 | 12.3 | 9.3 | 8.8 | 26.7 | 19.2 | 9.2 | 14.4 | 12.6 | 9.1 | 8.7 |
| C1 | 26.8 | 17.3 | 10.5 | 15.7 | 13.1 | 9.1 | 7.5 | 27.0 | 17.5 | 10.6 | 15.3 | 13.2 | 8.9 | 7.5 |
| C2 | 30.0 | 17.5 | 10.5 | 14.5 | 12.7 | 7.9 | 6.9 | 31.1 | 17.2 | 10.0 | 14.4 | 12.7 | 7.7 | 6.9 |
| D | 30.2 | 16.1 | 10.9 | 14.7 | 14.4 | 7.1 | 6.6 | 31.6 | 15.4 | 10.6 | 15.1 | 13.9 | 6.7 | 6.7 |
| E _Lowest_ | 34.6 | 13.5 | 8.6 | 14.3 | 15.0 | 7.0 | 7.1 | 34.9 | 13.5 | 8.4 | 14.5 | 15.0 | 6.8 | 6.9 |
| **Employment status** |  |  |  |  |  |  |  |  |  |  |  |  |  |  |
| Paid work | 26.8 | 17.2 | 11.1 | 15.6 | 13.1 | 8.7 | 7.6 | 26.7 | 17.1 | 11.0 | 15.4 | 13.2 | 9.9 | 7.8 |
| Student | 23.5 | 18.9 | 14.8 | 15.6 | 11.9 | 8.6 | 6.8 | 23.2 | 17.9 | 15.2 | 16.8 | 11.7 | 8.1 | 7.1 |
| Not in paid work | 30.5 | 14.7 | 8.8 | 15.2 | 15.4 | 7.8 | 7.7 | 30.9 | 14.5 | 8.6 | 15.5 | 15.1 | 7.8 | 7.6 |
| Retired | 41.1 | 18.3 | 6.8 | 10.9 | 12.4 | 5.3 | 5.1 | 41.8 | 17.9 | 6.5 | 11.1 | 12.5 | 5.0 | 5.2 |
| Missing | 38.1 | 9.3 | 15.4 | 5.6 | 10.8 | 13.0 | 7.8 | 37.3 | 11.9 | 15.3 | 6.8 | 10.2 | 11.9 | 6.8 |
| **Housing tenure** |  |  |  |  |  |  |  |  |  |  |  |  |  |  |
| Owner occupied | 29.8 | 17.5 | 8.6 | 16.1 | 12.7 | 8.2 | 7.1 | 30.7 | 17.6 | 8.3 | 15.7 | 12.7 | 7.9 | 7.1 |
| Private rented | 29.8 | 15.6 | 11.8 | 16.9 | 11.2 | 7.2 | 7.6 | 29.2 | 15.4 | 12.2 | 17.0 | 11.2 | 7.4 | 7.7 |
| Social rented | 32.9 | 13.8 | 7.8 | 16.0 | 14.2 | 7.7 | 7.5 | 33.6 | 13.6 | 7.6 | 15.9 | 14.0 | 7.5 | 7.7 |
| Other | 30.0 | 1.0 | 12.0 | 20.2 | 12.1 | 9.2 | 6.5 | 29.3 | 10.2 | 11.3 | 20.7 | 12.5 | 8.6 | 7.4 |
| Missing | 25.7 | 19.6 | 12.4 | 10.6 | 15.7 | 9.0 | 7.0 | 26.3 | 19.6 | 11.9 | 10.6 | 15.8 | 9.0 | 6.8 |
| **Education level** |  |  |  |  |  |  |  |  |  |  |  |  |  |  |
| University education | 24.3 | 17.8 | 10.3 | 16.2 | 13.3 | 9.0 | 9.0 | 24.5 | 17.7 | 10.1 | 15.8 | 13.6 | 9.2 | 9.1 |
| A-level or equivalent | 25.2 | 17.7 | 11.6 | 15.9 | 13.1 | 8.9 | 7.6 | 25.5 | 17.7 | 11.3 | 16.3 | 12.8 | 8.7 | 7.7 |
| GCSE/Vocational | 28.8 | 17.0 | 10.4 | 14.7 | 13.9 | 8.4 | 6.8 | 29.4 | 16.8 | 10.1 | 14.7 | 14.0 | 8.1 | 6.9 |
| No post-16 qualification | 40.8 | 14.5 | 8.0 | 12.6 | 13.3 | 5.5 | 5.3 | 41.3 | 14.3 | 8.0 | 12.4 | 13.1 | 5.4 | 5.7 |
| Other/still studying ^b^ | 32.8 | 15.6 | 9.4 | 14.2 | 12.7 | 8.0 | 7.3 | 33.4 | 15.5 | 9.0 | 14.2 | 12.9 | 7.9 | 7.1 |
| Missing | 35.9 | 17.2 | 10.2 | 10.3 | 16.1 | 3.6 | 6.7 | 36.8 | 16.1 | 9.7 | 11.0 | 15.1 | 4.4 | 7.0 |
| **Annual household income** |  |  |  |  |  |  |  |  |  |  |  |  |  |  |
| £50 000+ | 22.7 | 17.6 | 9.0 | 19.7 | 12.5 | 9.8 | 8.8 | 23.4 | 17.4 | 9.1 | 19.0 | 12.8 | 9.6 | 8.8 |
| £25 000–49 999 | 27.4 | 17.4 | 9.8 | 17.0 | 11.7 | 8.6 | 8.0 | 27.4 | 17.7 | 9.5 | 17.4 | 11.5 | 8.5 | 8.0 |
| £13 500–24 999 | 27.6 | 16.6 | 10.2 | 17.3 | 13.2 | 8.0 | 7.2 | 28.0 | 16.6 | 10.3 | 17.1 | 12.9 | 7.9 | 7.3 |
| up to £13 499 | 32.6 | 15.5 | 8.2 | 16.0 | 12.5 | 7.2 | 7.9 | 32.7 | 15.6 | 8.2 | 15.8 | 12.5 | 7.2 | 8.0 |
| Missing | 30.0 | 17.0 | 10.8 | 13.2 | 14.2 | 8.0 | 6.8 | 30.7 | 16.6 | 10.5 | 13.2 | 14.3 | 7.9 | 6.8 |
| ***Motivation To Stop Scale (MTSS) response categories:***   1. I don’t want to stop smoking *(Interpretation: absence of any belief, desire or intention)* 2. I think I should stop smoking but don’t really want to 3. I want to stop smoking but haven’t thought about when 4. I REALLY want to stop smoking but I don’t know when I will 5. I want to stop smoking and hope to soon 6. I REALLY want to stop smoking and intend to in the next 3 months 7. I REALLY want to stop smoking and intend to in the next month *(Interpretation: strong desire and short-term intention)*   ***MTSS****: Motivation To Stop Scale* | | | | | | | | | | | | | | |

# Level of tobacco addiction by socioeconomic position

## Research question 3

Among past-year smokers, to what extent does the level of tobacco addiction (measured by strength of urges to smoke) differ by socioeconomic position after adjusting for age, gender, and survey year?

## Table S8: Level of tobacco addiction (strength of urges to smoke) by socioeconomic position among past-year smokers (weighted dataset)

| **SEP indicator** | **N^a^** | **Strength of urges to smoke^b^, Mean** | **β [95%CI]^c^** | **β_adj_ [95%CI]^c^** |
| --- | --- | --- | --- | --- |
| ***Occupational social grade*** |  |  |  |  |
| AB | 5029 | 1.57 | *ref* | *ref* |
| C1 | 11399 | 1.61 | 0.04 [0.00, 0.08] | 0.06 [0.02, 0.11] |
| C2 | 7709 | 1.73 | 0.17 [0.12, 0.21] | 0.18 [0.14, 0.22] |
| D | 5855 | 1.80 | 0.24 [0.19, 0.29] | 0.26 [0.21, 0.31] |
| E | 5645 | 2.07 | 0.51 [0.46, 0.56] | 0.49 [0.44, 0.54] |
|  |  |  |  |  |
| ***Employment status*** |  |  |  |  |
| Paid work | 19789 | 1.64 | *ref* | *ref* |
| Student | 2011 | 1.34 | -0.31 [-0.36, -0.25] | -0.19 [-0.25, -0.12] |
| Not in paid work | 8194 | 2.02 | 0.38 [0.35, 0.42] | 0.36 [0.32, 0.39] |
| Retired | 5583 | 1.87 | 0.22 [0.19, 0.26] | 0.06 [-0.01, 0.12] |
| Missing | 60 | 1.71 | - | - |
|  |  |  |  |  |
| ***Housing tenure*** |  |  |  |  |
| Owner occupied | 10670 | 1.74 | *ref* | *ref* |
| Private rented | 7617 | 1.66 | -0.08 [-0.12, -0.04] | 0.03 [-0.01, 0.07] |
| Social rented | 7881 | 2.02 | 0.28 [0.24, 0.31] | 0.30 [0.27, 0.34] |
| Other | 287 | 1.66 | -0.08 [-0.23, 0.08] | -0.00 [-0.15, 0.15] |
| Missing | 9182 | 1.61 | - | - |
|  |  |  |  |  |
| ***Education*** |  |  |  |  |
| University degree | 8041 | 1.50 | *ref* | *ref* |
| A-level/equivalent | 7616 | 1.64 | 0.14 [0.10, 0.18] | 0.19 [0.14, 0.23] |
| GCSE/Vocational | 11250 | 1.84 | 0.34 [0.30, 0.38] | 0.33 [0.29, 0.37] |
| No post-16 qualification | 5913 | 2.02 | 0.52 [0.48, 0.56] | 0.46 [0.41, 0.50] |
| Other/still studying | 2507 | 1.72 | 0.22 [0.17, 0.28] | 0.22 [0.16, 0.28] |
| Missing | 310 | 1.83 | - | - |
|  |  |  |  |  |
| ***Household income*** |  |  |  |  |
| £50 000+ | 2032 | 1.57 | *ref* | *ref* |
| £25 000–49 999 | 4254 | 1.72 | 0.15 [0.09, 0.21] | 0.16 [0.10, 0.23] |
| £13 500–24 999 | 3558 | 1.87 | 0.30 [0.24, 0.37] | 0.31 [0.25, 0.38] |
| up to £13 499 | 5306 | 1.96 | 0.39 [0.33, 0.45] | 0.40 [0.34, 0.46] |
| Missing | 20487 | 1.69 | - | - |
| CI, confidence interval. **β**_adj_, effect adjusted for age, gender, and survey year. SEP, socioeconomic position.  ^a^ Unweighted sample size  ^b^ Strength of urges to smoke (level of tobacco addiction) scale: 0 (not at all) to 5 (extremely strong)  ^c^ weighted dataset (refer to supplementary Table S9 for unweighted dataset means and effect estimates). | | | | |

## Table S9: Level of tobacco addiction (strength of urges to smoke) by socioeconomic position (unweighted dataset)

| **SEP indicator** | **N** | **Strength of urges to smoke^1^, Mean** | **β [95%CI]** | **β_adj_ [95%CI]** |
| --- | --- | --- | --- | --- |
| ***Occupational social grade*** |  |  |  |  |
| AB | 5029 | 1.58 | *ref* | *ref* |
| C1 | 11399 | 1.61 | 0.03 [-0.01, 0.07] | 0.07 [0.03, 0.11] |
| C2 | 7709 | 1.76 | 0.18 [0.14, 0.22] | 0.18 [0.14, 0.22] |
| D | 5855 | 1.83 | 0.25 [0.21, 0.30] | 0.26 [0.22, 0.31] |
| E | 5645 | 2.09 | 0.51 [0.46, 0.55] | 0.48 [0.44, 0.53] |
|  |  |  |  |  |
| ***Employment status*** |  |  |  |  |
| Paid work | 19789 | 1.64 | *ref* | *ref* |
| Student | 2011 | 1.34 | -0.30 [-0.36, -0.25] | -0.18 [-0.24, -0.12] |
| Not in paid work | 8194 | 2.04 | 0.40 [0.37, 0.43] | 0.37 [0.34, 0.40] |
| Retired | 5583 | 1.87 | 0.23 [0.19, 0.27] | 0.08 [0.02, 0.14] |
| Missing | 60 | 1.68 | - | - |
|  |  |  |  |  |
| ***Housing tenure*** |  |  |  |  |
| Owner occupied | 10670 | 1.74 | *ref* | *ref* |
| Private rented | 7617 | 1.66 | -0.09 [-0.12, -0.05] | 0.03 [0.00, 0.07] |
| Social rented | 7881 | 2.02 | 0.28 [0.25, 0.31] | 0.30 [0.27, 0.34] |
| Other | 287 | 1.69 | -0.05 [-0.19, 0.08] | 0.03 [-0.11, 0.17] |
| Missing | 9182 | 1.61 | - | - |
|  |  |  |  |  |
| ***Education*** |  |  |  |  |
| University degree | 8041 | 1.51 | *ref* | *ref* |
| A-level/equivalent | 7616 | 1.64 | 0.13 [0.10, 0.17] | 0.17 [0.13, 0.21] |
| GCSE/Vocational | 11250 | 1.85 | 0.35 [0.31, 0.38] | 0.33 [0.29, 0.36] |
| No post-16 qualification | 5913 | 2.04 | 0.53 [0.49, 0.57] | 0.46 [0.42, 0.50] |
| Other/still studying | 2507 | 1.75 | 0.24 [0.19, 0.30] | 0.23 [0.18, 0.28] |
| Missing | 310 | 1.88 | - | - |
|  |  |  |  |  |
| ***Household income*** |  |  |  |  |
| £50 000+ | 2032 | 1.57 | *ref* | *ref* |
| £25 000–49 999 | 4254 | 1.73 | 0.16 [0.10, 0.22] | 0.17 [0.11, 0.23] |
| £13 500–24 999 | 3558 | 1.87 | 0.30 [0.23, 0.36] | 0.31 [0.25, 0.37] |
| up to £13 499 | 5306 | 1.95 | 0.38 [0.32, 0.44] | 0.40 [0.34, 0.46] |
| Missing | 20487 | 1.70 | - | - |
| CI, confidence interval. **β**_adj_, effect adjusted for age, gender, and survey year. SEP, socioeconomic position.  ^1^ Strength of urges to smoke (level of tobacco addiction) scale: 0 (not at all) to 5 (extremely strong) | | | | |

## Table S10: Level of tobacco addiction (strength of urges to smoke) by each SES subcategories

|  | **Percentage by strength of urges to smoke response categories by SES subcategories** | | | | | | | | | | | |
| --- | --- | --- | --- | --- | --- | --- | --- | --- | --- | --- | --- | --- |
| **SEP indicator** | **Weighted dataset** | | | | | | **Unweighted dataset** | | | | | |
|  | **0** | **1** | **2** | **3** | **4** | **5** | **0** | **1** | **2** | **3** | **4** | **5** |
| **Occupational social grade** |  |  |  |  |  |  |  |  |  |  |  |  |
| AB *_highest_* | 24.8 | 19.2 | 37.8 | 12.3 | 4.3 | 1.5 | 24.6 | 18.9 | 38.0 | 12.8 | 4.2 | 1.7 |
| C1 | 23.3 | 18.7 | 39.4 | 13.1 | 3.4 | 1.8 | 23.1 | 18.7 | 39.3 | 13.3 | 3.8 | 1.8 |
| C2 | 19.0 | 18.2 | 41.5 | 15.1 | 4.0 | 2.1 | 18.4 | 17.7 | 41.8 | 15.7 | 4.1 | 2.2 |
| D | 16.8 | 17.3 | 43.5 | 15.5 | 4.8 | 2.1 | 16.3 | 16.5 | 43.8 | 16.3 | 4.8 | 2.2 |
| E _Lowest_ | 12.8 | 14.8 | 40.3 | 20.3 | 7.9 | 3.9 | 12.6 | 14.5 | 40.2 | 20.8 | 7.9 | 4.0 |
| **Employment status** |  |  |  |  |  |  |  |  |  |  |  |  |
| Paid work | 21.7 | 18.7 | 40.7 | 13.3 | 3.8 | 1.8 | 21.9 | 18.6 | 40.4 | 13.5 | 3.8 | 1.8 |
| Student | 31.7 | 22.6 | 31.8 | 9.8 | 3.0 | 1.2 | 31.8 | 21.8 | 32.5 | 10.0 | 2.7 | 11.9 |
| Not in paid work | 13.7 | 15.0 | 41.0 | 19.3 | 7.5 | 3.6 | 13.4 | 14.7 | 40.9 | 19.9 | 7.5 | 3.7 |
| Retired | 15.5 | 16.3 | 43.5 | 17.5 | 5.0 | 2.2 | 15.4 | 16.2 | 43.7 | 17.5 | 4.9 | 2.3 |
| Missing | 28.1 | 13.2 | 36.4 | 11.3 | 4.2 | 6.9 | 28.3 | 13.3 | 35.0 | 13.3 | 5.0 | 5.0 |
| **Housing tenure** |  |  |  |  |  |  |  |  |  |  |  |  |
| Owner occupied | 18.8 | 16.9 | 43.3 | 15.3 | 4.1 | 1.7 | 18.7 | 16.9 | 43.1 | 15.6 | 3.9 | 1.7 |
| Private rented | 20.9 | 18.6 | 41.1 | 13.9 | 3.8 | 1.6 | 21.5 | 18.1 | 40.8 | 14.1 | 3.9 | 1.6 |
| Social rented | 12.8 | 14.8 | 43.2 | 19.3 | 6.9 | 3.0 | 12.8 | 14.8 | 43.0 | 19.5 | 6.9 | 3.2 |
| Other | 23.0 | 19.6 | 38.1 | 10.6 | 4.5 | 4.1 | 24.0 | 18.1 | 36.9 | 11.5 | 4.5 | 4.9 |
| Missing | 24.2 | 20.2 | 35.6 | 12.7 | 4.7 | 2.6 | 24.4 | 20.0 | 35.4 | 12.9 | 4.6 | 2.6 |
| **Education level** |  |  |  |  |  |  |  |  |  |  |  |  |
| University education | 27.4 | 19.2 | 36.4 | 11.8 | 3.6 | 1.6 | 27.3 | 19.1 | 36.2 | 12.2 | 3.6 | 1.7 |
| A-level or equivalent | 21.4 | 19.7 | 39.5 | 13.5 | 4.2 | 1.6 | 21.8 | 19.4 | 39.5 | 13.6 | 4.1 | 1.7 |
| GCSE/Vocational | 16.1 | 17.1 | 43.2 | 16.1 | 5.2 | 2.3 | 16.0 | 16.7 | 43.1 | 16.6 | 5.3 | 2.4 |
| No post-16 qualification | 13.2 | 14.4 | 43.1 | 19.6 | 6.1 | 3.6 | 12.8 | 14.3 | 43.0 | 20.1 | 6.3 | 3.6 |
| Other/still studying ^b^ | 19.5 | 18.5 | 41.3 | 14.0 | 4.6 | 2.2 | 19.1 | 18.2 | 40.9 | 14.8 | 4.6 | 2.5 |
| Missing | 21.0 | 15.7 | 37.1 | 16.6 | 4.9 | 4.7 | 19.7 | 15.2 | 38.4 | 16.1 | 5.5 | 5.2 |
| **Annual household income** |  |  |  |  |  |  |  |  |  |  |  |  |
| £50 000+ | 23.9 | 17.0 | 42.6 | 12.4 | 3.2 | 1.0 | 24.2 | 16.8 | 41.8 | 12.8 | 3.1 | 1.2 |
| £25 000–49 999 | 18.5 | 16.9 | 45.4 | 13.8 | 4.0 | 1.4 | 18.3 | 16.7 | 45.3 | 14.4 | 3.9 | 1.5 |
| £13 500–24 999 | 15.0 | 16.1 | 45.1 | 16.4 | 5.6 | 1.9 | 15.0 | 15.8 | 45.4 | 16.6 | 5.4 | 1.9 |
| up to £13 499 | 13.6 | 15.3 | 43.7 | 18.9 | 5.9 | 2.5 | 14.0 | 15.5 | 43.0 | 19.0 | 5.9 | 2.6 |
| Missing | 21.6 | 19.0 | 38.0 | 14.4 | 4.6 | 2.5 | 21.5 | 18.6 | 38.0 | 14.8 | 4.7 | 2.5 |
| ***Strength of urges to smoke response categories:***  *Question: How much of the time have you felt the urge to smoke in the past 24 hours?*   1. Not at all   *Question:* ‘*In general, how strong have the urges to smoke been?*   1. Slight 2. Moderate 3. Strong 4. Very strong 5. Extremely strong | | | | | | | | | | | | |

# Quit attempts by socioeconomic position

## Research question 4

Among past-year smokers, to what extent does the rate of past-year quit attempts differ by socioeconomic position after adjusting for age, gender, and survey year?

## Table S11: Quit attempts by socioeconomic position among past-year smokers (weighted dataset)

| **SEP indicator** | **N^a^** | **Percentage of respondents who made a quit attempt by SEP subcategories, %^b^** | **OR [95%CI]^b^** | **OR_adj_ [95%CI]^b^** |
| --- | --- | --- | --- | --- |
| ***Occupational social grade*** |  |  |  |  |
| AB | 4916 | 36.3 | *ref* | *ref* |
| C1 | 11205 | 35.5 | 0.96 [0.89, 1.04] | 0.92 [0.85, 0.99] |
| C2 | 7549 | 33.0 | 0.86 [0.80, 0.93] | 0.84 [0.78, 0.91] |
| D | 5713 | 33.2 | 0.87 [0.80, 0.95] | 0.83 [0.76, 0.91] |
| E | 5570 | 32.4 | 0.84 [0.77, 0.92] | 0.85 [0.78, 0.93] |
|  |  |  |  |  |
| ***Employment status*** |  |  |  |  |
| Paid work | 19381 | 35.1 | *ref* | *ref* |
| Student | 1960 | 39.0 | 1.19 [1.07, 1.31] | 1.02 [0.91, 1.15] |
| Not in paid work | 8058 | 35.7 | 1.03 [0.97, 1.09] | 1.03 [0.97, 1.10] |
| Retired | 5496 | 25.0 | 0.62 [0.57, 0.66] | 0.97 [0.86, 1.11] |
| Missing | 58 | 33.0 | - | - |
|  |  |  |  |  |
| ***Housing tenure*** |  |  |  |  |
| Owner occupied | 10398 | 31.6 | *ref* | *ref* |
| Private rented | 7434 | 32.7 | 1.06 [0.99, 1.13] | 0.90 [0.84, 0.97] |
| Social rented | 7708 | 34.2 | 1.13 [1.06, 1.21] | 1.05 [0.98, 1.12] |
| Other | 276 | 42.9 | 1.63 [1.25, 2.12] | 1.53 [1.17, 1.99] |
| Missing | 9137 | 37.7 |  |  |
|  |  |  |  |  |
| ***Education*** |  |  |  |  |
| University degree | 7837 | 37.9 | *ref* | *ref* |
| A-level/equivalent | 7514 | 37.0 | 0.96 [0.90, 1.03] | 0.93 [0.86, 1.00] |
| GCSE/Vocational | 11019 | 33.7 | 0.83 [0.78, 0.89] | 0.85 [0.79, 0.90] |
| No post-16 qualification | 5813 | 27.0 | 0.60 [0.56, 0.66] | 0.71 [0.65, 0.77] |
| Other/still studying | 2470 | 30.7 | 0.72 [0.65, 0.81] | 0.77 [0.69, 0.86] |
| Missing | 300 | 27.2 | - | - |
|  |  |  |  |  |
| ***Household income*** |  |  |  |  |
| £50 000+ | 1990 | 37.5 | *ref* | *ref* |
| £25 000–49 999 | 4166 | 34.2 | 0.86 [0.77, 0.97] | 0.86 [0.77, 0.97] |
| £13 500–24 999 | 3473 | 33.8 | 0.85 [0.75, 0.96] | 0.84 [0.74, 0.95] |
| up to £13 499 | 5172 | 33.0 | 0.82 [0.73, 0.92] | 0.83 [0.73, 0.93] |
| Missing | 20152 | 34.0 | - | - |
| CI, confidence interval. OR, odds ratio. OR_adj_, odds ratio adjusted for age, gender, and survey year. SEP, socioeconomic position.  ^a^ Unweighted sample size.  ^b^ weighted dataset (refer to supplementary Table S12 for unweighted dataset percentages and effect estimates). | | | | |

## Table S12: Quit attempts by socioeconomic position in past-year smokers (unweighted dataset)

| **SEP indicator** | **N** | **Percentage of respondents who made a quit attempt by SEP subcategories, %** | **OR [95%CI]** | **OR_adj_ [95%CI]** |
| --- | --- | --- | --- | --- |
| ***Occupational social grade*** |  |  |  |  |
| AB | 4916 | 35.4 | *ref* | *ref* |
| C1 | 11205 | 35.2 | 0.99 [0.93, 1.07] | 0.940.88, 1.01] |
| C2 | 7549 | 32.5 | 0.88 [0.82, 0.95] | 0.870.80, 0.94] |
| D | 5713 | 32.1 | 0.87 [0.80, 0.94] | 0.830.77, 0.90] |
| E | 5570 | 31.7 | 0.85 [0.78, 0.92] | 0.860.79, 0.93] |
|  |  |  |  |  |
| ***Employment status*** |  |  |  |  |
| Paid work | 19381 | 35.0 | *ref* | *ref* |
| Student | 1960 | 38.2 | 1.15 1.04, 1.26] | 1.000.90, 1.11] |
| Not in paid work | 8058 | 35.1 | 1.00 [0.95, 1.06] | 1.010.96, 1.07] |
| Retired | 5496 | 24.5 | 0.60 [0.56, 0.65] | 0.930.83, 1.05] |
| Missing | 58 | 29.3 | - | - |
|  |  |  |  |  |
| ***Housing tenure*** |  |  |  |  |
| Owner occupied | 10398 | 30.9 | *ref* | *ref* |
| Private rented | 7434 | 32.8 | 1.09 [1.02, 1.16] | 0.910.85, 0.97] |
| Social rented | 7708 | 33.4 | 1.12 [1.06, 1.20] | 1.040.97, 1.11] |
| Other | 276 | 42.4 | 1.65 [1.29, 2.09] | 1.521.18, 1.94] |
| Missing | 9137 | 37.1 | - | - |
|  |  |  |  |  |
| ***Education*** |  |  |  |  |
| University degree | 7837 | 37.4 | *ref* | *ref* |
| A-level/equivalent | 7514 | 36.9 | 0.98 [0.92, 1.04] | 0.950.88, 1.01] |
| GCSE/Vocational | 11019 | 33.5 | 0.84 [0.79, 0.89] | 0.860.81, 0.91] |
| No post-16 qualification | 5813 | 26.3 | 0.60 [0.55, 0.64] | 0.71 [0.66, 0.77] |
| Other/still studying | 2470 | 29.8 | 0.71 [0.64, 0.78] | 0.77 [0.69, 0.85] |
| Missing | 300 | 28.0 | - | - |
|  |  |  |  |  |
| ***Household income*** |  |  |  |  |
| £50 000+ | 1990 | 37.2 | *ref* | *ref* |
| £25 000–49 999 | 4166 | 33.9 | 0.87 [0.78, 0.97] | 0.87 [0.78, 0.97] |
| £13 500–24 999 | 3473 | 33.6 | 0.85 [0.76, 0.96] | 0.85 [0.76, 0.96] |
| up to £13 499 | 5172 | 32.4 | 0.81 [0.73, 0.90] | 0.82 [0.74, 0.92] |
| Missing | 20152 | 33.5 | - | - |
| CI, confidence interval. OR, odds ratio. OR_adj_, odds ratio adjusted for age, gender, and survey year. SEP, socioeconomic position.  - not applicable | | | | |

# Use of cessation aids by socioeconomic position

## Research question 5

Among past-year smokers who made a quit attempt, to what extent does the use of cessation aids and quitting success differ by socioeconomic position after adjusting for age, gender, survey year and level of tobacco addiction?

## Results for a selection of cessation aids

### Prescription nicotine replacement therapy

The odds of using prescription NRT were higher with increasing occupational social grade when compared with the most advantaged occupational social grade, however some estimates were imprecise (Table S21). Similarly, those not in paid work or living in local authority housing were more likely to use prescription NRT compared with people in the most advantaged socioeconomic category for each of these measures (Table S21). Respondents categorised as ‘other or still studying’ had higher odds of using prescription NRT than those who had attended university. Those on lower incomes were generally more likely to use prescription NRT when compared to the highest household income quartile, however, these estimates were inconclusive, and included the possibility of no difference and of favouring the opposite direction.

### Bupropion

There was inconclusive evidence that use of bupropion differed by any measure of socioeconomic position (Table S23). Directions of effect suggested greater odds of bupropion use for respondents who were students, not in paid work or retired compared to those in paid work, and lower odds of bupropion use when measured by housing tenure, education and household income, however all effect estimates were inconclusive.

### Varenicline

Relative to those in paid work, respondents who were retired or not in paid work had lower odds of using varenicline. There was no evidence of a difference in the use of varenicline for any of the other measures of socioeconomic position.

Directions of effect suggested that the odds of using varenicline were lower for most of the more disadvantaged relative to the most advantaged socioeconomic categories, as measured by occupational social grade, housing tenure, education level and household income after adjusting for age, gender, survey year and strength of urges to smoke (Table S25).

### Telephone support

Relative to those in the most advantaged occupational social grade, those in the two most disadvantaged social grade categories had higher odds of using telephone support. Similarly, people with no post-16 qualification also had higher odds of using telephone support than people with a university education level (Table S27).

Evidence on use of telephone support by employment status, housing tenure and household income were inconclusive. Directions of effect for all these favoured more disadvantaged groups, except for retired respondents for which the direction of effect favoured those in paid work.

### Written self-help materials

When compared to respondents who attended university, every other education category (excluding other/still studying) had lower odds of using self-help materials. Similarly, respondents from the lowest income bracket had lower odds of using self-help cessation aids compared with those in the highest household income bracket.

There was no evidence that the use of self-help materials differed by occupational social grade, employment status or housing tenure after adjusting for covariates however, the direction of effect mostly showed lower odds in more disadvantaged socioeconomic groups (Table S29).

### Alternative treatments

Respondents in the most disadvantaged occupational social grade had lower odds of using alternative treatments compared with those in the most advantaged social grade. This pattern was also seen for the other social grade categories and by income level and education, however estimates were inconclusive.

Those not in paid work had lower odds of using alternative cessation aids than those in paid work. Those living in local authority or association housing had lower odds than homeowners of using alternative treatments (Table S31).

## Table S13: Use of over the counter NRT by socioeconomic position (weighted dataset)

| **SEP indicator** | **N^a^** | **Percentage of respondents who used the cessation aid by SES subcategories, %^b^** | **OR [95% CI]^b^** | **OR_adj_ [95%CI]^b^** |
| --- | --- | --- | --- | --- |
| ***Occupational social grade*** |  |  |  |  |
| AB | 1738 | 18.2 | *ref* | *ref* |
| C1 | 3943 | 16.5 | 0.90 [0.77, 1.05] | 0.95 [0.81, 1.12] |
| C2 | 2456 | 17.9 | 0.96 [0.81, 1.13] | 0.96 [0.81, 1.14] |
| D | 1835 | 17.5 | 0.95 [0.79, 1.15] | 0.97 [0.80, 1.17] |
| E | 1767 | 21.8 | 1.26 [1.06, 1.50] | 1.15 [0.96, 1.37] |
| ***Employment status*** |  |  |  |  |
| Paid work | 6792 | 16.6 | *ref* | *ref* |
| Student | 749 | 12.5 | 0.72 [0.56, 0.92] | 1.07 [0.81, 1.40] |
| Not in paid work | 2832 | 19.1 | 1.19 [1.05, 1.34] | 1.08 [0.95, 1.22] |
| Retired | 1349 | 24.9 | 1.66 [1.44, 1.93] | 1.53 [1.16, 2.02] |
| Missing | 17 | - | - | - |
| ***Housing tenure*** |  |  |  |  |
| Owner occupied | 3213 | 19.0 | *ref* | *ref* |
| Private rented | 2437 | 16.4 | 0.84 [0.72, 0.97] | 0.99 [0.85, 1.17] |
| Social rented | 2578 | 19.2 | 1.01 [0.88, 1.17] | 0.98 [0.84, 1.13] |
| Other | 117 | 16.3 | 0.83 [0.49, 1.40] | 1.08 [0.63, 1.83] |
| Missing | 3394 | - | - | - |
| ***Education*** |  |  |  |  |
| University degree | 2931 | 17.3 | *ref* | *ref* |
| A-level/equivalent | 2771 | 16.8 | 0.96 [0.83, 1.12] | 1.02 [0.88, 1.19] |
| GCSE/Vocational | 3689 | 18.7 | 1.10 [0.96, 1.26] | 1.04 [0.90, 1.20] |
| No post-16 qualification | 1528 | 18.6 | 1.09 [0.92, 1.30] | 0.87 [0.72, 1.04] |
| Other/still studying | 736 | 15.6 | 0.88 [0.69, 1.12] | 0.87 [0.68, 1.11] |
| Missing | 84 | - | - | - |
| ***Household income*** |  |  |  |  |
| £50 000+ | 740 | 15.1 | *ref* | *ref* |
| £25 000–49 999 | 1414 | 18.6 | 1.29 [1.00, 1.67] | 1.31 [1.01, 1.70] |
| £13 500–24 999 | 1166 | 20.4 | 1.44 [1.11, 1.87] | 1.36 [1.04, 1.78] |
| up to £13 499 | 1674 | 19.7 | 1.38 [1.07, 1.77] | 1.31 [1.01, 1.70] |
| Missing | 6745 | - | - | - |
| CI, confidence interval. OR, odds ratio. OR_adj_, odds ratio adjusted for age, gender, survey year and level of tobacco addiction. SEP, socioeconomic position.  ^a^ Unweighted sample size.  ^b^ weighted dataset (refer to supplementary Table S14 for unweighted dataset percentages and effect estimates). | | | | |

## Table S14: Use of over the counter NRT by socioeconomic position (unweighted dataset)

| **SEP indicator** | **N** | **Percentage of respondents who used the cessation aid by SES subcategories, %** | **OR [95% CI]** | **OR_adj_ [95%CI]** |
| --- | --- | --- | --- | --- |
| ***Occupational social grade*** |  |  |  |  |
| AB | 1738 | 18.2 | *ref* | *ref* |
| C1 | 3943 | 16.5 | 0.89 [0.77, 1.03] | 0.96 [0.83, 1.12] |
| C2 | 2456 | 17.9 | 0.98 [0.83, 1.15] | 0.97 [0.83, 1.14] |
| D | 1835 | 17.5 | 0.95 [0.80, 1.13] | 0.95 [0.80, 1.13] |
| E | 1767 | 21.8 | 1.25 [1.06, 1.48] | 1.14 [0.96, 1.35] |
| ***Employment status*** |  |  |  |  |
| Paid work | 6792 | 16.7 | *ref* | *ref* |
| Student | 749 | 12.1 | 0.69 [0.55, 0.86] | 1.04 [0.81, 1.34] |
| Not in paid work | 2832 | 19.4 | 1.20 [1.07, 1.34] | 1.07 [0.95, 1.20] |
| Retired | 1349 | 25.1 | 1.67 [1.45, 1.91] | 1.60 [1.26, 2.03] |
| Missing | 17 | - | - | - |
| ***Housing tenure*** |  |  |  |  |
| Owner occupied | 3213 | 19.5 | *ref* | *ref* |
| Private rented | 2437 | 16.5 | 0.82 [0.71, 0.94] | 1.01 [0.87, 1.17] |
| Social rented | 2578 | 19.5 | 1.00 [0.88, 1.14] | 0.96 [0.84, 1.10] |
| Other | 117 | 17.1 | 0.85 [0.51, 1.36] | 1.11 [0.66, 1.80] |
| Missing | 3394 | - | - | - |
| ***Education*** |  |  |  |  |
| University degree | 2931 | 17.4 | *ref* | *ref* |
| A-level/equivalent | 2771 | 17.1 | 0.98 [0.85, 1.12] | 1.04 [0.90, 1.19] |
| GCSE/Vocational | 3689 | 18.9 | 1.10 [0.97, 1.25] | 1.03 [0.90, 1.17] |
| No post-16 qualification | 1528 | 19.8 | 1.17 [1.00, 1.37] | 0.90 [0.76, 1.07] |
| Other/still studying | 736 | 15.8 | 0.89 [0.71, 1.10] | 0.86 [0.69, 1.07] |
| Missing | 84 | - |  |  |
| ***Household income*** |  |  |  |  |
| £50 000+ | 740 | 15.1 | *ref* | *ref* |
| £25 000–49 999 | 1414 | 18.5 | 1.27 [1.00, 1.62] | 1.26 [0.99, 1.61] |
| £13 500–24 999 | 1166 | 20.9 | 1.48 [1.16, 1.90] | 1.37 [1.06, 1.76] |
| up to £13 499 | 1674 | 19.4 | 1.35 [1.07, 1.71] | 1.25 [0.98, 1.60] |
| Missing | 6745 | - | - | - |
| CI, confidence interval. OR, odds ratio. OR_adj_, odds ratio adjusted for age, gender, survey year and level of tobacco addiction. SEP, socioeconomic position. | | | | |

## Table S15: Use of electronic cigarettes by socioeconomic position (weighted dataset)

| **SEP indicator** | **N^a^** | **Percentage of respondents who used the cessation aid by SES subcategories, %^b^** | **OR [95% CI]^b^** | **OR_adj_ [95%CI]^b^** |
| --- | --- | --- | --- | --- |
| ***Occupational social grade*** |  |  |  |  |
| AB | 1738 | 32.5 | *ref* | *ref* |
| C1 | 3943 | 30.7 | 0.92 [0.81, 1.05] | 0.91 [0.80, 1.04] |
| C2 | 2456 | 37.3 | 1.23 [1.07, 1.42] | 1.21 [1.06, 1.39] |
| D | 1835 | 33.7 | 1.06 [0.91, 1.23] | 1.02 [0.87, 1.19] |
| E | 1767 | 29.8 | 0.88 [0.76, 1.03] | 0.85 [0.73, 1.00] |
| ***Employment status*** |  |  |  |  |
| Paid work | 6792 | 35.4 | *ref* | *ref* |
| Student | 749 | 27.2 | 0.68 [0.57, 0.82] | 0.65 [0.54, 0.80] |
| Not in paid work | 2832 | 32.2 | 0.87 [0.78, 0.96] | 0.84 [0.75, 0.93] |
| Retired | 1349 | 22.8 | 0.54 [0.46, 0.62] | 0.70 [0.54, 0.89] |
| Missing | 17 | - | - | - |
| ***Housing tenure*** |  |  |  |  |
| Owner occupied | 3213 | 34.4 | *ref* | *ref* |
| Private rented | 2437 | 33.4 | 0.95 [0.85, 1.08] | 0.88 [0.78, 1.00] |
| Social rented | 2578 | 34.7 | 1.01 [0.90, 1.14] | 0.95 [0.84, 1.07] |
| Other | 117 | 36.2 | 1.08 [0.71, 1.63] | 1.04 [0.68, 1.59] |
| Missing | 3394 | - | - | - |
| ***Education*** |  |  |  |  |
| University degree | 2931 | 29.0 | *ref* | *ref* |
| A-level/equivalent | 2771 | 34.0 | 1.26 [1.12, 1.43] | 1.26 [1.11, 1.43] |
| GCSE/Vocational | 3689 | 36.8 | 1.43 [1.27, 1.60] | 1.42 [1.26, 1.60] |
| No post-16 qualification | 1528 | 30.7 | 1.09 [0.94, 1.26] | 1.23 [1.05, 1.44] |
| Other/still studying | 736 | 32.1 | 1.16 [0.96, 1.40] | 1.20 [0.98, 1.45] |
| Missing | 84 | - | - | - |
| ***Household income*** |  |  |  |  |
| £50 000+ | 740 | 36.6 | *ref* | *ref* |
| £25 000–49 999 | 1414 | 35.5 | 0.95 [0.78, 1.16] | 0.97 [0.80, 1.19] |
| £13 500–24 999 | 1166 | 33.8 | 0.88 [0.72, 1.09] | 0.91 [0.73, 1.12] |
| up to £13 499 | 1674 | 31.8 | 0.81 [0.66, 0.98] | 0.85 [0.69, 1.04] |
| Missing | 6745 | - | - | - |
| CI, confidence interval. OR, odds ratio. OR_adj_, odds ratio adjusted for age, gender, survey year and level of tobacco addiction. SEP, socioeconomic position.  ^a^ Unweighted sample size.  ^b^ weighted dataset (refer to supplementary Table S16 for unweighted dataset percentages and effect estimates). | | | | |

## Table S16: Use of electronic cigarettes by socioeconomic position (unweighted dataset)

| **SEP indicator** | **N** | **Percentage of respondents who used the cessation aid by SES subcategories, %** | **OR [95% CI]** | **OR_adj_ [95%CI]** |
| --- | --- | --- | --- | --- |
| ***Occupational social grade*** |  |  |  |  |
| AB | 1738 | 32.0 | *ref* | *ref* |
| C1 | 3943 | 30.3 | 0.93 [0.82, 1.05] | 0.91 [0.80, 1.03] |
| C2 | 2456 | 35.9 | 1.19 [1.05, 1.36] | 1.17 [1.03, 1.34] |
| D | 1835 | 33.7 | 1.08 [0.94, 1.24] | 1.04 [0.90, 1.20] |
| E | 1767 | 29.5 | 0.89 [0.77, 1.03] | 0.86 [0.74, 0.99] |
| ***Employment status*** |  |  |  |  |
| Paid work | 6792 | 34.7 | *ref* | *ref* |
| Student | 749 | 26.8 | 0.69 [0.58, 0.82] | 0.66 [0.55, 0.80] |
| Not in paid work | 2832 | 32.1 | 0.89 [0.81, 0.98] | 0.86 [0.78, 0.94] |
| Retired | 1349 | 22.6 | 0.55 [0.48, 0.63] | 0.71 [0.56, 0.89] |
| Missing | 17 | - | - | - |
| ***Housing tenure*** |  |  |  |  |
| Owner occupied | 3213 | 33.3 | *ref* | *ref* |
| Private rented | 2437 | 32.3 | 0.96 [0.86, 1.07] | 0.87 [0.77, 0.97] |
| Social rented | 2578 | 33.4 | 1.01 [0.90, 1.12] | 0.93 [0.83, 1.05] |
| Other | 117 | 37.6 | 1.21 [0.82, 1.76] | 1.14 [0.77, 1.67] |
| Missing | 3394 | - | - | - |
| ***Education*** |  |  |  |  |
| University degree | 2931 | 28.3 | *ref* | *ref* |
| A-level/equivalent | 2771 | 33.1 | 1.25 [1.12, 1.40] | 1.25 [1.11, 1.40] |
| GCSE/Vocational | 3689 | 35.9 | 1.42 [1.28, 1.58] | 1.43 [1.28, 1.59] |
| No post-16 qualification | 1528 | 29.8 | 1.08 [0.94, 1.23] | 1.23 [1.06, 1.42] |
| Other/still studying | 736 | 31.1 | 1.15 [0.96, 1.36] | 1.19 [1.00, 1.42] |
| Missing | 84 | - | - | - |
| ***Household income*** |  |  |  |  |
| £50 000+ | 740 | 35.8 | *ref* | *ref* |
| £25 000–49 999 | 1414 | 34.9 | 0.96 [0.80, 1.16] | 0.98 [0.81, 1.18] |
| £13 500–24 999 | 1166 | 32.7 | 0.87 [0.72, 1.06] | 0.89 [0.73, 1.09] |
| up to £13 499 | 1674 | 30.8 | 0.80 [0.66, 0.96] | 0.83 [0.69, 1.01] |
| Missing | 6745 | - | - | - |
| CI, confidence interval. OR, odds ratio. OR_adj_, odds ratio adjusted for age, gender, survey year and level of tobacco addiction. SEP, socioeconomic position. | | | | |

## Table S17: Use of prescription pharmacotherapies (NRT with prescription, Bupropion and Varenicline) by socioeconomic position (weighted dataset)

| **SEP indicator** | **N^a^** | **Percentage of respondents who used the cessation aid by SES subcategories, %^b^** | **OR [95% CI]^b^** | **OR_adj_ [95%CI]^b^** |
| --- | --- | --- | --- | --- |
| ***Occupational social grade*** |  |  |  |  |
| AB | 1738 | 8.2 | *ref* | *ref* |
| C1 | 3943 | 7.0 | 0.85 [0.68, 1.06] | 0.96 [0.76, 1.20] |
| C2 | 2456 | 8.0 | 0.98 [0.77, 1.24] | 1.02 [0.80, 1.29] |
| D | 1835 | 8.6 | 1.05 [0.82, 1.35] | 1.15 [0.89, 1.49] |
| E | 1767 | 9.7 | 1.20 [0.94, 1.54] | 1.07 [0.83, 1.38] |
| ***Employment status*** |  |  |  |  |
| Paid work | 6792 | 7.4 | *ref* | *ref* |
| Student | 749 | 2.7 | 0.35 [0.21, 0.56] | 0.91 [0.54, 1.53] |
| Not in paid work | 2832 | 9.8 | 1.38 [1.17, 1.62] | 1.16 [0.97, 1.38] |
| Retired | 1349 | 12.3 | 1.76 [1.44, 2.16] | 0.86 [0.61, 1.20] |
| Missing | 17 | - | - | - |
| ***Housing tenure*** |  |  |  |  |
| Owner occupied | 3213 | 8.8 | *ref* | *ref* |
| Private rented | 2437 | 6.4 | 0.70 [0.56, 0.87] | 0.95 [0.75, 1.20] |
| Social rented | 2578 | 10.7 | 1.17 [0.97, 1.42] | 1.21 [1.00, 1.47] |
| Other | 117 | 5.1 | 0.57 [0.24, 1.37] | 0.99 [0.41, 2.39] |
| Missing | 3394 | - | - | - |
| ***Education*** |  |  |  |  |
| University degree | 2931 | 7.2 | *ref* | *ref* |
| A-level/equivalent | 2771 | 6.9 | 0.95 [0.77, 1.18] | 1.12 [0.90, 1.40] |
| GCSE/Vocational | 3689 | 8.4 | 1.17 [0.97, 1.43] | 1.07 [0.88, 1.32] |
| No post-16 qualification | 1528 | 10.1 | 1.44 [1.14, 1.83] | 1.02 [0.79, 1.30] |
| Other/still studying | 736 | 10.7 | 1.53 [1.12, 2.09] | 1.53 [1.11, 2.11] |
| Missing | 84 |  |  |  |
| ***Household income*** |  |  |  |  |
| £50 000+ | 740 | 8.0 | *ref* | *ref* |
| £25 000–49 999 | 1414 | 10.5 | 1.36 [0.97, 1.91] | 1.39 [0.98, 1.96] |
| £13 500–24 999 | 1166 | 9.8 | 1.25 [0.88, 1.78] | 1.13 [0.79, 1.62] |
| up to £13 499 | 1674 | 10.4 | 1.34 [0.97, 1.86] | 1.22 [0.87, 1.71] |
| Missing | 6745 | - | - | - |
| CI, confidence interval. OR, odds ratio. OR_adj_, odds ratio adjusted for age, gender, survey year and level of tobacco addiction. SEP, socioeconomic position.  ^a^ Unweighted sample size.  ^b^ weighted dataset (refer to supplementary Table S18 for unweighted dataset percentages and effect estimates). | | | | |

## Table S18: Use of prescription pharmacotherapies (NRT with prescription, Bupropion and Varenicline) by socioeconomic position (unweighted dataset)

| **SEP indicator** | **N** | **Percentage of respondents who used the cessation aid by SES subcategories, %** | **OR [95% CI]** | **OR_adj_ [95%CI]** |
| --- | --- | --- | --- | --- |
| ***Occupational social grade*** |  |  |  |  |
| AB | 1738 | 8.2 | *ref* | *ref* |
| C1 | 3943 | 6.8 | 0.81 [0.66, 1.00] | 0.94 [0.76, 1.17] |
| C2 | 2456 | 8.3 | 1.01 [0.81, 1.26] | 1.01 [0.81, 1.27] |
| D | 1835 | 8.8 | 1.08 [0.85, 1.37] | 1.10 [0.87, 1.40] |
| E | 1767 | 1.0 | 1.23 [0.98, 1.56] | 1.08 [0.86, 1.37] |
| ***Employment status*** |  |  |  |  |
| Paid work | 6792 | 7.2 | *ref* | *ref* |
| Student | 749 | 2.5 | 0.34 [0.21, 0.52] | 0.85 [0.50, 1.35] |
| Not in paid work | 2832 | 10.0 | 1.43 [1.23, 1.67] | 1.19 [1.02, 1.40] |
| Retired | 1349 | 12.3 | 1.80 [1.49, 2.16] | 0.89 [0.64, 1.22] |
| Missing | 17 | - | - | - |
| ***Housing tenure*** |  |  |  |  |
| Owner occupied | 3213 | 9.1 | *ref* | *ref* |
| Private rented | 2437 | 6.6 | 0.70 [0.57, 0.86] | 0.97 [0.78, 1.21] |
| Social rented | 2578 | 10.5 | 1.24 [1.04, 1.47] | 1.25 [1.04, 1.49] |
| Other | 117 | 5.4 | 0.56 [0.22, 1.18] | 0.94 [0.36, 2.02] |
| Missing | 3394 | - | - | - |
| ***Education*** |  |  |  |  |
| University degree | 2931 | 7.2 | *ref* | *ref* |
| A-level/equivalent | 2771 | 7.0 | 0.97 [0.79, 1.18] | 1.12 [0.91, 1.37] |
| GCSE/Vocational | 3689 | 8.4 | 1.18 [0.98, 1.41] | 1.03 [0.86, 1.25] |
| No post-16 qualification | 1528 | 10.3 | 1.47 [1.18, 1.82] | 0.99 [0.79, 1.24] |
| Other/still studying | 736 | 10.0 | 1.37 [1.03, 1.81] | 1.32 [0.98, 1.75] |
| Missing | 84 | - | - | - |
| ***Household income*** |  |  |  |  |
| £50 000+ | 740 | 8.1 | *ref* | *ref* |
| £25 000–49 999 | 1414 | 10.3 | 1.29 [0.95, 1.79] | 1.28 [0.94, 1.78] |
| £13 500–24 999 | 1166 | 9.6 | 1.20 [0.87, 1.68] | 1.06 [0.76, 1.49] |
| up to £13 499 | 1674 | 10.3 | 1.31 [0.97, 1.79] | 1.16 [0.85, 1.60] |
| Missing | 6745 | - | - | - |
| CI, confidence interval. OR, odds ratio. OR_adj_, odds ratio adjusted for age, gender, survey year and level of tobacco addiction. SEP, socioeconomic position. | | | | |

## Table S19: Use of face-to-face behavioural support by socioeconomic position (weighted dataset)

| **SEP indicator** | **N^a^** | **Percentage of respondents who used the cessation aid by SES subcategories, %^b^** | **OR [95% CI]^b^** | **OR_adj_ [95%CI]^b^** |
| --- | --- | --- | --- | --- |
| ***Occupational social grade*** |  |  |  |  |
| AB | 1738 | 2.9 | *ref* | *ref* |
| C1 | 3943 | 2.0 | 0.67 [0.46, 0.97] | 0.71 [0.49, 1.03] |
| C2 | 2456 | 2.8 | 0.94 [0.64, 1.37] | 0.97 [0.66, 1.42] |
| D | 1835 | 2.7 | 0.92 [0.60, 1.40] | 0.92 [0.60, 1.42] |
| E | 1767 | 3.0 | 1.03 [0.68, 1.55] | 0.86 [0.57, 1.32] |
| ***Employment status*** |  |  |  |  |
| Paid work | 6792 | 2.2 | *ref* | *ref* |
| Student | 749 | 0.8 | 0.35 [0.12, 0.99] | 0.61 [0.19, 1.89] |
| Not in paid work | 2832 | 3.3 | 1.50 [1.13, 1.99] | 1.31 [0.98, 1.76] |
| Retired | 1349 | 4.6 | 2.13 [1.53, 2.96] | 0.98 [0.45, 2.14] |
| Missing | 17 | - | - | - |
| ***Housing tenure*** |  |  |  |  |
| Owner occupied | 3213 | 2.8 | *ref* | ***ref*** |
| Private rented | 2437 | 2.0 | 0.71 [0.49, 1.03] | 0.99 [0.67, 1.46] |
| Social rented | 2578 | 3.2 | 1.14 [0.82, 1.58] | 1.11 [0.80, 1.55] |
| Other | 117 | 2.4 | 0.84 [0.26, 2.73] | 1.33 [0.41, 4.35] |
| Missing | 3394 |  |  |  |
| ***Education*** |  |  |  |  |
| University degree | 2931 | 2.6 | *ref* | *ref* |
| A-level/equivalent | 2771 | 2.1 | 0.77 [0.53, 1.13] | 0.81 [0.55, 1.21] |
| GCSE/Vocational | 3689 | 2.9 | 1.09 [0.79, 1.49] | 1.00 [0.72, 1.38] |
| No post-16 qualification | 1528 | 3.5 | 1.33 [0.90, 1.96] | 0.92 [0.61, 1.38] |
| Other/still studying | 736 | 1.6 | 0.59 [0.30, 1.14] | 0.57 [0.29, 1.12] |
| Missing | 84 | - | - | - |
| ***Household income*** |  |  |  |  |
| £50 000+ | 740 | 1.7 | *ref* | *ref* |
| £25 000–49 999 | 1414 | 2.1 | 1.28 [0.64, 2.53] | 1.27 [0.63, 2.54] |
| £13 500–24 999 | 1166 | 4.1 | 2.56 [1.34, 4.90] | 2.27 [1.15, 4.45] |
| up to £13 499 | 1674 | 3.6 | 2.22 [1.17, 4.19] | 1.87 [0.97, 3.62] |
| Missing | 6745 | - | - | - |
| CI, confidence interval. OR, odds ratio. OR_adj_, odds ratio adjusted for age, gender, survey year and level of tobacco addiction. SEP, socioeconomic position.  ^a^ Unweighted sample size.  ^b^ weighted dataset (refer to supplementary Table S20 for unweighted dataset percentages and effect estimates). | | | | |

## Table S20: Use of face-to-face behavioural support by socioeconomic position (unweighted dataset)

| **SEP indicator** | **N** | **Percentage of respondents who used the cessation aid by SES subcategories, %** | **OR [95% CI]** | **OR_adj_ [95%CI]** |
| --- | --- | --- | --- | --- |
| ***Occupational social grade*** |  |  |  |  |
| AB | 1738 | 3.1 | *ref* | *ref* |
| C1 | 3943 | 2.0 | 0.66 [0.46, 0.94] | 0.72 [0.51, 1.03] |
| C2 | 2456 | 3.0 | 0.99 [0.69, 1.42] | 0.99 [0.70, 1.43] |
| D | 1835 | 2.7 | 0.89 [0.60, 1.32] | 0.88 [0.59, 1.31] |
| E | 1767 | 2.9 | 0.94 [0.64, 1.40] | 0.80 [0.54, 1.19] |
| ***Employment status*** |  |  |  |  |
| Paid work | 6792 | 2.3 | *ref* | *ref* |
| Student | 749 | 0.7 | 0.28 [0.10, 0.62] | 0.50 [0.17, 1.14] |
| Not in paid work | 2832 | 3.1 | 1.37 [1.05, 1.78] | 1.19 [0.91, 1.56] |
| Retired | 1349 | 4.2 | 1.86 [1.36, 2.52] | 0.86 [0.50, 1.48] |
| Missing | 17 | - | - | - |
| ***Housing tenure*** |  |  |  |  |
| Owner occupied | 3213 | 3.0 | *ref* | *ref* |
| Private rented | 2437 | 2.0 | 0.65 [0.46, 0.92] | 0.92 [0.63, 1.33] |
| Social rented | 2578 | 3.1 | 1.04 [0.77, 1.40] | 1.02 [0.74, 1.39] |
| Other | 117 | 2.6 | 0.85 [0.21, 2.32] | 1.28 [0.31, 3.54] |
| Missing | 3394 | - | - | - |
| ***Education*** |  |  |  |  |
| University degree | 2931 | 2.7 | *ref* | *ref* |
| A-level/equivalent | 2771 | 2.0 | 0.74 [0.52, 1.05] | 0.78 [0.54, 1.11] |
| GCSE/Vocational | 3689 | 3.0 | 1.15 [0.86, 1.54] | 1.03 [0.76, 1.40] |
| No post-16 qualification | 1528 | 3.3 | 1.26 [0.88, 1.80] | 0.86 [0.59, 1.26] |
| Other/still studying | 736 | 1.5 | 0.55 [0.28, 1.00] | 0.53 [0.26, 0.97] |
| Missing | 84 | - | - | - |
| ***Household income*** |  |  |  |  |
| £50 000+ | 740 | 1.8 | *ref* | *ref* |
| £25 000–49 999 | 1414 | 2.2 | 1.25 [0.67, 2.50] | 1.21 [0.64, 2.40] |
| £13 500–24 999 | 1166 | 4.0 | 2.35 [1.30, 4.55] | 2.05 [1.12, 3.98] |
| up to £13 499 | 1674 | 3.4 | 1.94 [1.09, 3.72] | 1.64 [0.91, 3.17] |
| Missing | 6745 | - | - | - |
| CI, confidence interval. OR, odds ratio. OR_adj_, odds ratio adjusted for age, gender, survey year and level of tobacco addiction. SEP, socioeconomic position. | | | | |

## Table S21: Use of prescription NRT by socioeconomic position (weighted dataset)

| **SEP indicator** | **N^a^** | **Percentage of respondents who used the cessation aid by SES subcategories, %^b^** | **OR [95% CI]^b^** | **OR_adj_ [95%CI]^b^** |
| --- | --- | --- | --- | --- |
| ***Occupational social grade*** |  |  |  |  |
| AB | 1738 | 3.8 | *ref* | *ref* |
| C1 | 3943 | 3.1 | 0.81 [0.59, 1.12] | 0.90 [0.65, 1.25] |
| C2 | 2456 | 3.8 | 1.00 [0.72, 1.39] | 1.03 [0.74, 1.43] |
| D | 1835 | 4.5 | 1.20 [0.84, 1.70] | 1.29 [0.91, 1.85] |
| E | 1767 | 6.1 | 1.67 [1.20, 2.31] | 1.48 [1.06, 2.06] |
| ***Employment status*** |  |  |  |  |
| Paid work | 6792 | 3.1 | *ref* | *ref* |
| Student | 749 | 1.6 | 0.49 [0.26, 0.92] | 1.26 [0.65, 2.46] |
| Not in paid work | 2832 | 5.9 | 1.97 [1.57, 2.47] | 1.75 [1.39, 2.21] |
| Retired | 1349 | 7.2 | 2.41 [1.84, 3.16] | 1.29 [0.87, 1.91] |
| Missing | 17 | - |  |  |
| ***Housing tenure*** |  |  |  |  |
| Owner occupied | 3213 | 3.9 | *ref* | *ref* |
| Private rented | 2437 | 3.2 | 0.81 [0.59, 1.12] | 1.14 [0.81, 1.59] |
| Social rented | 2578 | 5.6 | 1.47 [1.13, 1.91] | 1.54 [1.17, 2.02] |
| Other | 117 | 1.6 | 0.40 [0.10, 1.66] | 0.64 [0.15, 2.67] |
| Missing | 3394 | - |  |  |
| ***Education*** |  |  |  |  |
| University degree | 2931 | 3.3 | *ref* | *ref* |
| A-level/equivalent | 2771 | 3.4 | 1.03 [0.76, 1.41] | 1.20 [0.87, 1.66] |
| GCSE/Vocational | 3689 | 4.1 | 1.26 [0.95, 1.66] | 1.17 [0.88, 1.57] |
| No post-16 qualification | 1528 | 6.1 | 1.92 [1.40, 2.62] | 1.35 [0.97, 1.88] |
| Other/still studying | 736 | 5.5 | 1.72 [1.10, 2.69] | 1.67 [1.06, 2.64] |
| Missing | 84 | - |  |  |
| ***Household income*** |  |  |  |  |
| £50 000+ | 740 | 3.0 | *ref* | *ref* |
| £25 000–49 999 | 1414 | 4.0 | 1.38 [0.81, 2.38] | 1.37 [0.79, 2.36] |
| £13 500–24 999 | 1166 | 4.4 | 1.53 [0.89, 2.61] | 1.37 [0.80, 2.36] |
| up to £13 499 | 1674 | 5.2 | 1.80 [1.09, 2.96] | 1.62 [0.97, 2.70] |
| Missing | 6745 | - |  |  |
| CI, confidence interval. OR, odds ratio. OR_adj_, odds ratio adjusted for age, gender, survey year and level of tobacco addiction. SEP, socioeconomic position.  ^a^ Unweighted sample size.  ^b^ weighted dataset (refer to supplementary Table S22 for unweighted dataset percentages and effect estimates). | | | | |

## Table S22: Use of prescription NRT by socioeconomic position (unweighted dataset)

| **SEP indicator** | **N** | **Percentage of respondents who used the cessation aid by SES subcategories, %** | **OR [95% CI]** | **OR_adj_ [95%CI]** |
| --- | --- | --- | --- | --- |
| ***Occupational social grade*** |  |  |  |  |
| AB | 1738 | 4.0 | *ref* | *ref* |
| C1 | 3943 | 3.0 | 0.74 [0.55, 1.01] | 0.84 [0.62, 1.14] |
| C2 | 2456 | 4.0 | 1.00 [0.73, 1.37] | 1.01 [0.74, 1.39] |
| D | 1835 | 4.6 | 1.14[ 0.83, 1.58] | 1.21 [0.87, 1.69] |
| E | 1767 | 6.1 | 1.54 [1.13, 2.10] | 1.38 [1.01, 1.89] |
| ***Employment status*** |  |  |  |  |
| Paid work | 6792 | 3.0 | *ref* | *ref* |
| Student | 749 | 1.5 | 0.48 [0.24, 0.84] | 1.23 [0.61, 2.28] |
| Not in paid work | 2832 | 6.0 | 2.04 [1.65, 2.51] | 1.81 [1.46, 2.25] |
| Retired | 1349 | 7.0 | 2.41 [1.86, 3.09] | 1.35 [0.88, 2.05] |
| Missing | 17 | - | - | - |
| ***Housing tenure*** |  |  |  |  |
| Owner occupied | 3213 | 3.8 | *ref* | *ref* |
| Private rented | 2437 | 3.0 | 0.77 [0.57, 1.03] | 1.13 [0.82, 1.54] |
| Social rented | 2578 | 5.8 | 1.57 [1.23, 2.00] | 1.62 [1.26, 2.09] |
| Other | 117 | 1.7 | 0.44 [0.07, 1.41] | 0.69 [0.11, 2.24] |
| Missing | 3394 | - | - | - |
| ***Education*** |  |  |  |  |
| University degree | 2931 | 3.2 | *ref* | *ref* |
| A-level/equivalent | 2771 | 3.5 | 1.08 [0.81, 1.45] | 1.26 [0.94, 1.68] |
| GCSE/Vocational | 3689 | 4.2 | 1.32 [1.02, 1.72] | 1.21 [0.93, 1.59] |
| No post-16 qualification | 1528 | 6.4 | 2.05 [1.53, 2.74] | 1.43 [1.05, 1.93] |
| Other/still studying | 736 | 4.8 | 1.51 [1.00, 2.22] | 1.44 [0.95, 2.12] |
| Missing | 84 | - | - | - |
| ***Household income*** |  |  |  |  |
| £50 000+ | 740 | 3.1 | *ref* | *ref* |
| £25 000–49 999 | 1414 | 3.8 | 1.24 [0.76, 2.07] | 1.20 [0.74, 2.03] |
| £13 500–24 999 | 1166 | 4.6 | 1.48 [0.91, 2.49] | 1.30 [0.79, 2.19] |
| up to £13 499 | 1674 | 5.3 | 1.75 [1.12, 2.86] | 1.54 [0.97, 2.55] |
| Missing | 6745 | - | - | - |
| CI, confidence interval. OR, odds ratio. OR_adj_, odds ratio adjusted for age, gender, survey year and level of tobacco addiction. SEP, socioeconomic position. | | | | |

## Table S23: Use of bupropion by socioeconomic position (weighted dataset)

| **SEP indicator** | **N^a^** | **Percentage of respondents who used the cessation aid by SES subcategories, %^b^** | **OR [95% CI]^b^** | **OR_adj_ [95%CI]^b^** |
| --- | --- | --- | --- | --- |
| ***Occupational social grade*** |  |  |  |  |
| AB | 1738 | 0.6 | *ref* | *ref* |
| C1 | 3943 | 0.6 | 0.99 [0.45, 2.21] | 1.11 [0.49, 2.51] |
| C2 | 2456 | 0.6 | 1.03 [0.45, 2.37] | 1.06 [0.47, 2.42] |
| D | 1835 | 0.4 | 0.71 [0.25, 2.02] | 0.74 [0.27, 2.03] |
| E | 1767 | 0.7 | 1.07 [0.45, 2.54] | 0.81 [0.34, 1.92] |
| ***Employment status*** |  |  |  |  |
| Paid work | 6792 | 0.4 | *ref* | *ref* |
| Student | 749 | 0.2 | 0.60 [0.14, 2.57] | 1.85 [0.39, 8.82] |
| Not in paid work | 2832 | 0.8 | 2.04 [1.08, 3.84] | 1.53 [0.78, 3.01] |
| Retired | 1349 | 1.4 | 3.48 [1.89, 6.42] | 1.56 [0.42, 5.82] |
| Missing | 17 | - | - | - |
| ***Housing tenure*** |  |  |  |  |
| Owner occupied | 3213 | 1.0 | *ref* | *ref* |
| Private rented | 2437 | 0.3 | 0.27 [0.10, 0.73] | 0.45 [0.16, 1.22] |
| Social rented | 2578 | 0.6 | 0.54 [0.29, 1.02] | 0.53 [0.27, 1.04] |
| Other^c^ | 117 | 0.0 | - | - |
| Missing | 3394 | - | - | - |
| ***Education*** |  |  |  |  |
| University degree | 2931 | 0.6 | *ref* | *ref* |
| A-level/equivalent | 2771 | 0.5 | 0.77 [0.36, 1.63] | 0.89 [0.43, 1.85] |
| GCSE/Vocational | 3689 | 0.5 | 0.87 [0.45, 1.68] | 0.76 [0.40, 1.42] |
| No post-16 qualification | 1528 | 0.5 | 0.78 [0.31, 1.97] | 0.42 [0.16, 1.12] |
| Other/still studying | 736 | 1.4 | 2.30 [0.89, 5.97] | 2.22 [0.82, 6.00] |
| Missing | 84 | - | - | - |
| ***Household income*** |  |  |  |  |
| £50 000+ | 740 | 0.8 | *ref* | *ref* |
| £25 000–49 999 | 1414 | 0.5 | 0.70 [0.21, 2.36] | 0.71 [0.21, 2.47] |
| £13 500–24 999 | 1166 | 0.8 | 1.0 [10.30, 3.40] | 0.76 [0.22, 2.55] |
| up to £13 499 | 1674 | 0.7 | 0.9 [20.31, 2.76] | 0.64 [0.21, 1.90] |
| Missing | 6745 | - | - | - |
| CI, confidence interval. OR, odds ratio. OR_adj_, odds ratio adjusted for age, gender, survey year and level of tobacco addiction. SEP, socioeconomic position.  ^a^ Unweighted sample size.  ^b^ weighted dataset (refer to supplementary Table S24 for unweighted dataset percentages and effect estimates).  ^c^ Reliable estimates could not be calculated due to no respondents in this category using this cessation aid (n=0) | | | | |

## Table S24: Use of bupropion by socioeconomic position (unweighted dataset)

| **SEP indicator** | **N** | **Percentage of respondents who used the cessation aid by SES subcategories, %** | **OR [95% CI]** | **OR_adj_ [95%CI]** |
| --- | --- | --- | --- | --- |
| ***Occupational social grade*** |  |  |  |  |
| AB | 1738 | 0.6 | *ref* | *ref* |
| C1 | 3943 | 0.6 | 0.96 [0.48, 2.05] | 1.14 [0.54, 2.54] |
| C2 | 2456 | 0.7 | 1.03 [0.48, 2.29] | 1.04 [0.47, 2.43] |
| D | 1835 | 0.4 | 0.60 [0.22, 1.53] | 0.62 [0.21, 1.67] |
| E | 1767 | 0.8 | 1.25 [0.57, 2.83] | 0.98 [0.42, 2.34] |
| ***Employment status*** |  |  |  |  |
| Paid work | 6792 | 0.4 | *ref* | *ref* |
| Student | 749 | 0.3 | 0.62 [0.10, 2.08] | 2.45 [0.37, 9.50] |
| Not in paid work | 2832 | 0.7 | 1.66 [0.92, 2.92] | 1.26 [0.69, 2.23] |
| Retired | 1349 | 1.6 | 3.69 [2.07, 6.46] | 1.66 [0.64, 4.23] |
| Missing | 17 | - | - | - |
| ***Housing tenure*** |  |  |  |  |
| Owner occupied | 3213 | 1.0 | *ref* | *ref* |
| Private rented | 2437 | 0.2 | 0.20 [0.07, 0.46] | 0.34 [0.11, 0.87] |
| Social rented | 2578 | 0.7 | 0.64 [0.35, 1.13] | 0.63 [0.33, 1.15] |
| Other | 117 | 0.0 | - | - |
| Missing | 3394 | - | - | - |
| ***Education*** |  |  |  |  |
| University degree | 2931 | 0.7 | *ref* | *ref* |
| A-level/equivalent | 2771 | 0.5 | 0.69 [0.33, 1.37] | 0.79 [0.37, 1.64] |
| GCSE/Vocational | 3689 | 0.6 | 0.91 [0.50, 1.68] | 0.77 [0.40, 1.47] |
| No post-16 qualification | 1528 | 0.5 | 0.77 [0.32, 1.68] | 0.38 [0.15, 0.89] |
| Other/still studying | 736 | 1.0 | 1.40 [0.55, 3.17] | 1.26 [0.46, 3.00] |
| Missing | 84 | - | - | - |
| ***Household income*** |  |  |  |  |
| £50 000+ | 740 | 0.8 | *ref* | *ref* |
| £25 000–49 999 | 1414 | 0.5 | 0.61 [0.20, 1.90] | 0.57 [0.19, 1.75] |
| £13 500–24 999 | 1166 | 0.7 | 0.85 [0.29, 2.58] | 0.58 [0.20, 1.78] |
| up to £13 499 | 1674 | 0.8 | 0.96 [0.38, 2.74] | 0.62 [0.23, 1.79] |
| Missing | 6745 | - | - | - |
| CI, confidence interval. OR, odds ratio. OR_adj_, odds ratio adjusted for age, gender, survey year and level of tobacco addiction. SEP, socioeconomic position.  a Reliable estimates could not be calculated due to no respondents in this category using this cessation aid (n=0)  - not applicable | | | | |

## Table S25: Use of varenicline by socioeconomic position (weighted dataset)

| **SEP indicator** | **N^a^** | **Percentage of respondents who used the cessation aid by SES subcategories, %^b^** | **OR [95% CI]^b^** | **OR_adj_ [95%CI]^b^** |
| --- | --- | --- | --- | --- |
| ***Occupational social grade*** |  |  |  |  |
| AB | 1738 | 4.3 | *ref* | *ref* |
| C1 | 3943 | 3.7 | 0.86 [0.63, 1.16] | 0.97 [0.71, 1.31] |
| C2 | 2456 | 4.2 | 0.98 [0.71, 1.35] | 1.03 [0.74, 1.43] |
| D | 1835 | 4.4 | 1.01 [0.71, 1.42] | 1.11 [0.78, 1.59] |
| E | 1767 | 3.5 | 0.81 [0.57, 1.16] | 0.74 [0.51, 1.06] |
| ***Employment status*** |  |  |  |  |
| Paid work | 6792 | 4.4 | *ref* | *ref* |
| Student | 749 | 1.0 | 0.23 [0.10, 0.50] | 0.55 [0.23, 1.30] |
| Not in paid work | 2832 | 3.6 | 0.83 [0.65, 1.06] | 0.68 [0.53, 0.87] |
| Retired | 1349 | 4.8 | 1.10 [0.82, 1.49] | 0.47 [0.27, 0.82] |
| Missing | 17 | - | - | - |
| ***Housing tenure*** |  |  |  |  |
| Owner occupied | 3213 | 4.4 | *ref* | *ref* |
| Private rented | 2437 | 1.0 | 0.65 [0.49, 0.88] | 0.84 [0.61, 1.16] |
| Social rented | 2578 | 3.6 | 0.94 [0.72, 1.22] | 0.95 [0.72, 1.26] |
| Other | 117 | 4.8 | 0.75 [0.25, 2.20] | 1.33 [0.45, 3.94] |
| Missing | 3394 | - | - | - |
| ***Education*** |  |  |  |  |
| University degree | 2931 | 4.0 | *ref* | *ref* |
| A-level/equivalent | 2771 | 3.4 | 0.86 [0.64, 1.16] | 1.01 [0.74, 1.36] |
| GCSE/Vocational | 3689 | 4.2 | 1.06 [0.81, 1.38] | 0.96 [0.73, 1.27] |
| No post-16 qualification | 1528 | 4.0 | 1.00 [0.71, 1.42] | 0.74 [0.51, 1.07] |
| Other/still studying | 736 | 5.6 | 1.43 [0.94, 2.19] | 1.46 [0.94, 2.27] |
| Missing | 84 | - | - | - |
| ***Household income*** |  |  |  |  |
| £50 000+ | 740 | 5.1 | *ref* | *ref* |
| £25 000–49 999 | 1414 | 6.7 | 1.32 [0.87, 2.02] | 1.37 [0.90, 2.10] |
| £13 500–24 999 | 1166 | 4.8 | 0.93 [0.59, 1.48] | 0.87 [0.55, 1.37] |
| up to £13 499 | 1674 | 5.0 | 0.96 [0.63, 1.48] | 0.91 [0.59, 1.42] |
| Missing | 6745 | - | - | - |
| CI, confidence interval. OR, odds ratio. OR_adj_, odds ratio adjusted for age, gender, survey year and level of tobacco addiction. SEP, socioeconomic position.  ^a^ Unweighted sample size.  ^b^ weighted dataset (refer to supplementary Table S26 for unweighted dataset percentages and effect estimates). | | | | |

## Table S26: Use of varenicline by socioeconomic position (unweighted dataset)

| **SEP indicator** | **N** | **Percentage of respondents who used the cessation aid by SES subcategories, %** | **OR [95% CI]** | **OR_adj_ [95%CI]** |
| --- | --- | --- | --- | --- |
| ***Occupational social grade*** |  |  |  |  |
| AB | 1738 | 4.2 | *ref* | *ref* |
| C1 | 3943 | 3.5 | 0.83 [0.63, 1.12] | 0.97 [0.73, 1.30] |
| C2 | 2456 | 4.2 | 0.99 [0.73, 1.35] | 0.98 [0.72, 1.33] |
| D | 1835 | 4.4 | 1.05 [0.76, 1.46] | 1.03 [0.74, 1.42] |
| E | 1767 | 3.8 | 0.90 [0.64, 1.26] | 0.78 [0.56, 1.10] |
| ***Employment status*** |  |  |  |  |
| Paid work | 6792 | 4.2 | *ref* | *ref* |
| Student | 749 | 0.9 | 0.22 [0.09, 0.42] | 0.48 [0.20, 0.98] |
| Not in paid work | 2832 | 3.8 | 0.91 [0.72, 1.14] | 0.72 [0.57, 0.91] |
| Retired | 1349 | 4.6 | 1.10 [0.83, 1.45] | 0.45 [0.28, 0.72] |
| Missing | 17 | - | - | - |
| ***Housing tenure*** |  |  |  |  |
| Owner occupied | 3213 | 4.2 | *ref* | *ref* |
| Private rented | 2437 | 0.9 | 0.70 [0.53, 0.92] | 0.89 [0.67, 1.19] |
| Social rented | 2578 | 3.8 | 0.97 [0.76, 1.24] | 0.95 [0.74, 1.22] |
| Other | 117 | 4.6 | 0.72 [0.22, 1.74] | 1.23 [0.38, 2.99] |
| Missing | 3394 | - | - | - |
| ***Education*** |  |  |  |  |
| University degree | 2931 | 4.0 | *ref* | *ref* |
| A-level/equivalent | 2771 | 3.3 | 0.85 [0.64, 1.12] | 0.96 [0.73, 1.27] |
| GCSE/Vocational | 3689 | 4.1 | 1.03 [0.80, 1.32] | 0.88 [0.69, 1.14] |
| No post-16 qualification | 1528 | 3.9 | 0.97 [0.70, 1.34] | 0.67 [0.48, 0.93] |
| Other/still studying | 736 | 5.0 | 1.28 [0.87, 1.86] | 1.25 [0.84, 1.80] |
| Missing | 84 | - | - | - |
| ***Household income*** |  |  |  |  |
| £50 000+ | 740 | 5.0 | *ref* | *ref* |
| £25 000–49 999 | 1414 | 6.4 | 1.29 [0.88, 1.94] | 1.30 [0.88, 1.95] |
| £13 500–24 999 | 1166 | 4.6 | 0.92 [0.60, 1.43] | 0.84 [0.54, 1.30] |
| up to £13 499 | 1674 | 4.7 | 0.94 [0.64, 1.42] | 0.86 [0.57, 1.32] |
| Missing | 6745 | - | - | - |
| CI, confidence interval. OR, odds ratio. OR_adj_, odds ratio adjusted for age, gender, survey year and level of tobacco addiction. SEP, socioeconomic position.  - not applicable | | | | |

## Table S27: Use of telephone support by socioeconomic position (weighted dataset)

| **SEP indicator** | **N^a^** | **Percentage of respondents who used the cessation aid by SES subcategories, %^b^** | **OR [95% CI]^b^** | **OR_adj_ [95%CI]^b^** |
| --- | --- | --- | --- | --- |
| ***Occupational social grade*** |  |  |  |  |
| AB | 1738 | 0.4 | *ref* | *ref* |
| C1 | 3943 | 0.5 | 1.50 [0.62, 3.66] | 1.47 [0.59, 3.66] |
| C2 | 2456 | 0.6 | 1.77 [0.69, 4.54] | 1.84 [0.71, 4.73] |
| D | 1835 | 1.1 | 3.00 [1.17, 7.69] | 2.92 [1.12, 7.65] |
| E | 1767 | 1.0 | 2.83 [1.14, 7.02] | 2.75 [1.09, 6.98] |
| ***Employment status*** |  |  |  |  |
| Paid work | 6792 | 0.7 | *ref* | *ref* |
| Student | 749 | 0.4 | 0.65 [0.19, 2.28] | 1.14 [0.30, 4.29] |
| Not in paid work | 2832 | 0.7 | 1.10 [0.60, 2.00] | 1.19 [0.66, 2.15] |
| Retired | 1349 | 1.1 | 1.70 [0.86, 3.35] | 0.91 [0.21, 4.07] |
| Missing | 17 | - | - | - |
| ***Housing tenure*** |  |  |  |  |
| Owner occupied | 3213 | 0.4 | *ref* | *ref* |
| Private rented | 2437 | 0.4 | 1.03 [0.43, 2.45] | 1.60 [0.62, 4.11] |
| Social rented | 2578 | 0.7 | 1.58 [0.71, 3.53] | 1.46 [0.63, 3.41] |
| Other^c^ | 117 | 0.0 | - | - |
| Missing | 3394 | - | - | - |
| ***Education*** |  |  |  |  |
| University degree | 2931 | 0.5 | *ref* | *ref* |
| A-level/equivalent | 2771 | 0.5 | 1.04 [0.48, 2.27] | 1.08 [0.48, 2.43] |
| GCSE/Vocational | 3689 | 0.8 | 1.64 [0.83, 3.24] | 2.06 [0.99, 4.29] |
| No post-16 qualification | 1528 | 1.2 | 2.59 [1.21, 5.51] | 2.89 [1.26, 6.60] |
| Other/still studying | 736 | 1.0 | 2.08 [0.62, 7.02] | 2.55 [0.74, 8.81] |
| Missing | 84 | - | - | - |
| ***Household income*** |  |  |  |  |
| £50 000+ | 740 | 7.1 | *ref* | *ref* |
| £25 000–49 999 | 1414 | 12.6 | 2.26 [0.51, 9.95] | 2.17 [0.51, 9.33] |
| £13 500–24 999 | 1166 | 9.7 | 1.86 [0.43, 8.08] | 1.80 [0.44, 7.32] |
| up to £13 499 | 1674 | 12.7 | 1.72 [0.42, 7.03] | 1.66 [0.39, 7.00] |
| Missing | 6745 | - |  |  |
| CI, confidence interval. OR, odds ratio. OR_adj_, odds ratio adjusted for age, gender, survey year and level of tobacco addiction. SEP, socioeconomic position.  ^a^ Unweighted sample size.  ^b^ weighted dataset (refer to supplementary Table S28 for unweighted dataset percentages and effect estimates).  ^c^ Reliable estimates could not be calculated due to no respondents in this category using this cessation aid (n=0) | | | | |

## Table S28: Use of telephone support by socioeconomic position (unweighted dataset)

| **SEP indicator** | **N** | **Percentage of respondents who used the cessation aid by SES subcategories, %** | **OR [95% CI]** | **OR_adj_ [95%CI]** |
| --- | --- | --- | --- | --- |
| ***Occupational social grade*** |  |  |  |  |
| AB | 1738 | 0.4 | *ref* | *ref* |
| C1 | 3943 | 0.6 | 1.39 [0.62, 3.52] | 1.34 [0.59, 3.48] |
| C2 | 2456 | 0.6 | 1.42 [0.59, 3.75] | 1.52 [0.62, 4.08] |
| D | 1835 | 0.8 | 2.04 [0.86, 5.35] | 2.19 [0.89, 5.91] |
| E | 1767 | 1.0 | 2.54 [1.11, 6.56] | 2.58 [1.10, 6.81] |
| ***Employment status*** |  |  |  |  |
| Paid work | 6792 | 0.7 | *ref* | *ref* |
| Student | 749 | 0.4 | 0.66 [0.16, 1.83] | 1.02 [0.23, 3.18] |
| Not in paid work | 2832 | 0.7 | 1.11 [0.63, 1.89] | 1.24 [0.69, 2.17] |
| Retired | 1349 | 1.0 | 1.60 [0.82, 2.92] | 1.25 [0.38, 4.01] |
| Missing | 17 | - | - | - |
| ***Housing tenure*** |  |  |  |  |
| Owner occupied | 3213 | 0.5 | *ref* | *ref* |
| Private rented | 2437 | 0.4 | 0.88 [0.38, 1.93] | 1.29 [0.53, 3.02] |
| Social rented | 2578 | 0.5 | 1.16 [0.56, 2.42] | 1.11 [0.50, 2.39] |
| Other^a^ | 117 | 0.0 | - | - |
| Missing | 3394 | - | - | - |
| ***Education*** |  |  |  |  |
| University degree | 2931 | 0.5 | *ref* | *ref* |
| A-level/equivalent | 2771 | 0.5 | 0.99 [0.47, 2.06] | 1.11 [0.51, 2.41] |
| GCSE/Vocational | 3689 | 0.7 | 1.38 [0.74, 2.67] | 1.74 [0.91, 3.48] |
| No post-16 qualification | 1528 | 1.1 | 2.06 [1.01, 4.21] | 2.39 [1.10, 5.24] |
| Other/still studying | 736 | 0.5 | 1.06 [0.30, 2.94] | 1.30 [0.36, 3.68] |
| Missing | 84 |  | - | - |
| ***Household income*** |  |  |  |  |
| £50 000+ | 740 | 0.3 | *ref* | *ref* |
| £25 000–49 999 | 1414 | 0.6 | 1.05 [0.28, 4.98] | 1.00 [0.26, 4.79] |
| £13 500–24 999 | 1166 | 0.5 | 1.27 [0.33, 6.04] | 1.19 [0.31, 5.76] |
| up to £13 499 | 1674 | 0.5 | 1.18 [0.34, 5.40] | 1.07 [0.30, 5.05] |
| Missing | 6745 | - | - | - |
| CI, confidence interval. OR, odds ratio. OR_adj_, odds ratio adjusted for age, gender, survey year and level of tobacco addiction. SEP, socioeconomic position.  ^a^ Reliable estimates could not be calculated due to no respondents in this category using this cessation aid (n=0)  - not applicable | | | | |

## Table S29: Use of written self-help materials by socioeconomic position (weighted dataset)

| **SEP indicator** | **N^a^** | **Percentage of respondents who used the cessation aid by SES subcategories, %^b^** | **OR [95% CI]^b^** | **OR_adj_ [95%CI]^b^** |
| --- | --- | --- | --- | --- |
| ***Occupational social grade*** |  |  |  |  |
| AB | 1738 | 5.8 | *ref* | *ref* |
| C1 | 3943 | 5.5 | 0.95 [0.74, 1.22] | 0.88 [0.68, 1.14] |
| C2 | 2456 | 5.4 | 0.93 [0.70, 1.23] | 0.90 [0.68, 1.20] |
| D | 1835 | 4.7 | 0.81 [0.59, 1.12] | 0.75 [0.54, 1.04] |
| E | 1767 | 4.5 | 0.78 [0.56, 1.07] | 0.76 [0.54, 1.05] |
| ***Employment status*** |  |  |  |  |
| Paid work | 6792 | 5.6 | *ref* | *ref* |
| Student | 749 | 7.7 | 1.41 [1.02, 1.95] | 1.41 [0.98, 2.03] |
| Not in paid work | 2832 | 4.6 | 0.81 [0.64, 1.02] | 0.80 [0.64, 1.01] |
| Retired | 1349 | 3.1 | 0.55 [0.39, 0.78] | 0.61 [0.34, 1.10] |
| Missing | 17 | - | - | - |
| ***Housing tenure*** |  |  |  |  |
| Owner occupied | 3213 | 4.4 | *ref* | *ref* |
| Private rented | 2437 | 5.5 | 1.26 [0.97, 1.63] | 1.14 [0.87, 1.51] |
| Social rented | 2578 | 3.9 | 0.88 [0.67, 1.17] | 0.78 [0.59, 1.04] |
| Other | 117 | 4.1 | 0.91 [0.34, 2.46] | 0.69 [0.23, 2.11] |
| Missing | 3394 | - | - | - |
| ***Education*** |  |  |  |  |
| University degree | 2931 | 7.3 | *ref* | *ref* |
| A-level/equivalent | 2771 | 6.0 | 0.80 [0.64, 1.01] | 0.77 [0.61, 0.97] |
| GCSE/Vocational | 3689 | 4.2 | 0.55 [0.44, 0.70] | 0.56 [0.44, 0.71] |
| No post-16 qualification | 1528 | 1.8 | 0.23 [0.15, 0.36] | 0.26 [0.16, 0.41] |
| Other/still studying | 736 | 5.9 | 0.79 [0.54, 1.15] | 0.83 [0.56, 1.21] |
| Missing | 84 |  | - | - |
| ***Household income*** |  |  |  |  |
| £50 000+ | 740 | 5.7 | *ref* | *ref* |
| £25 000–49 999 | 1414 | 4.3 | 0.73 [0.48, 1.12] | 0.72 [0.47, 1.10] |
| £13 500–24 999 | 1166 | 5.4 | 0.94 [0.62, 1.42] | 0.89 [0.58, 1.36] |
| up to £13 499 | 1674 | 3.9 | 0.67 [0.45, 1.02] | 0.64 [0.43, 0.97] |
| Missing | 6745 | - |  |  |
| CI, confidence interval. OR, odds ratio. OR_adj_, odds ratio adjusted for age, gender, survey year and level of tobacco addiction. SEP, socioeconomic position.  ^a^ Unweighted sample size.  ^b^ weighted dataset (refer to supplementary Table S30 for unweighted dataset percentages and effect estimates). | | | | |

## Table S30: Use of written self-help materials by socioeconomic position (unweighted dataset)

| **SEP indicator** | **N** | **Percentage of respondents who used the cessation aid by SES subcategories, %** | **OR [95% CI]** | **OR_adj_ [95%CI]** |  |
| --- | --- | --- | --- | --- | --- |
| ***Occupational social grade*** |  |  |  |  |  |
| AB | 1738 | 5.7 | *ref* | *ref* |  |
| C1 | 3943 | 5.5 | 0.96 [0.76, 1.24] | 0.90 [0.70, 1.15] |  |
| C2 | 2456 | 5.0 | 0.87 [0.67, 1.15] | 0.87 [0.66, 1.15] |  |
| D | 1835 | 4.3 | 0.74 [0.55, 1.01] | 0.73 [0.53, 0.99] |  |
| E | 1767 | 4.1 | 0.70 [0.51, 0.96] | 0.70 [0.51, 0.96] |  |
| ***Employment status*** |  |  |  |  |  |
| Paid work | 6792 | 5.6 | *ref* | *ref* |  |
| Student | 749 | 7.3 | 1.34 [0.99, 1.78] | 1.38 [0.99, 1.90] |  |
| Not in paid work | 2832 | 4.1 | 0.71 [0.57, 0.88] | 0.73 [0.58, 0.90] |  |
| Retired | 1349 | 3.0 | 0.52 [0.36, 0.71] | 0.64 [0.35, 1.13] |  |
| Missing | 17 | - | - | - |  |
| ***Housing tenure*** |  |  |  |  |  |
| Owner occupied | 3213 | 4.5 | *ref* | *ref* |  |
| Private rented | 2437 | 5.3 | 1.21 [0.95, 1.54] | 1.07 [0.83, 1.39] |  |
| Social rented | 2578 | 3.8 | 0.86 [0.66, 1.11] | 0.78 [0.60, 1.02] |  |
| Other | 117 | 4.3 | 0.96 [0.33, 2.16] | 0.66 [0.20, 1.61] |  |
| Missing | 3394 | - | - | - |  |
| ***Education*** |  |  |  |  |  |
| University degree | 2931 | 7.2 | *ref* | *ref* |  |
| A-level/equivalent | 2771 | 5.6 | 0.76 [0.61, 0.94] | 0.74 [0.59, 0.92] |  |
| GCSE/Vocational | 3689 | 4.1 | 0.55 [0.44, 0.68] | 0.56 [0.45, 0.70] |  |
| No post-16 qualification | 1528 | 1.7 | 0.22 [0.14, 0.33] | 0.26 [0.16, 0.38] |  |
| Other/still studying | 736 | 5.7 | 0.78 [0.54, 1.08] | 0.83 [0.58, 1.16] |  |
| Missing | 84 | - | - | - |  |
| ***Household income*** |  |  |  |  |  |
| £50 000+ | 740 | 6.0 | *ref* | *ref* |  |
| £25 000–49 999 | 1414 | 4.5 | 0.74 [0.50, 1.10] | 0.73 [0.49, 1.09] |  |
| £13 500–24 999 | 1166 | 5.2 | 0.87 [0.59, 1.31] | 0.85 [0.57, 1.28] |  |
| up to £13 499 | 1674 | 3.8 | 0.63 [0.43, 0.94] | 0.61 [0.41, 0.93] |  |
| Missing | 6745 | - | - | - |  |
| CI, confidence interval. OR, odds ratio. OR_adj_, odds ratio adjusted for age, gender, survey year and level of tobacco addiction. SEP, socioeconomic position.  - not applicable | | | | | |

## Table S31: Use of alternative treatments by socioeconomic position (weighted dataset)

| **SEP indicator** | **N^a^** | **Percentage of respondents who used the cessation aid by SES subcategories, %^b^** | **OR [95% CI]^b^** | **OR_adj_ [95%CI]^b^** |
| --- | --- | --- | --- | --- |
| ***Occupational social grade*** |  |  |  |  |
| AB | 1738 | 1.4 | *ref* | *ref* |
| C1 | 3943 | 1.1 | 0.80 [0.48, 1.35] | 1.00 [0.58, 1.74] |
| C2 | 2456 | 1.0 | 0.68 [0.37, 1.25] | 0.75 [0.40, 1.41] |
| D | 1835 | 1.1 | 0.78 [0.40, 1.51] | 0.95 [0.48, 1.90] |
| E | 1767 | 0.6 | 0.39 [0.18, 0.85] | 0.34 [0.15, 0.80] |
| ***Employment status*** |  |  |  |  |
| Paid work | 6792 | 1.1 | *ref* | *ref* |
| Student | 749 | 0.9 | 0.79 [0.33, 1.93] | 2.32 [0.99, 5.41] |
| Not in paid work | 2832 | 0.7 | 0.64 [0.38, 1.08] | 0.47 [0.27, 0.84] |
| Retired | 1349 | 1.5 | 1.31 [0.75, 2.28] | 0.38 [0.14, 1.00] |
| Missing | 17 | - | - | - |
| ***Housing tenure*** |  |  |  |  |
| Owner occupied | 3213 | 1.6 | *ref* | *ref* |
| Private rented | 2437 | 0.7 | 0.44 [0.24, 0.80] | 0.63 [0.34, 1.16] |
| Social rented | 2578 | 0.7 | 0.42 [0.23, 0.78] | 0.45 [0.25, 0.81] |
| Other | 117 | 0.6 | 0.39 [0.05, 2.89] | 0.60 [0.08, 4.46] |
| Missing | 3394 | - | - | - |
| ***Education*** |  |  |  |  |
| University degree | 2931 | 1.2 | *ref* | *ref* |
| A-level/equivalent | 2771 | 1.1 | 0.95 [0.54, 1.66] | 1.13 [0.63, 2.03] |
| GCSE/Vocational | 3689 | 0.9 | 0.80 [0.47, 1.35] | 0.73 [0.41, 1.28] |
| No post-16 qualification | 1528 | 1.0 | 0.86 [0.43, 1.70] | 0.57 [0.28, 1.16] |
| Other/still studying | 736 | 1.1 | 0.97 [0.41, 2.29] | 0.95 [0.39, 2.30] |
| Missing | 84 | - | - | - |
| ***Household income*** |  |  |  |  |
| £50 000+ | 740 | 1.7 | *ref* | *ref* |
| £25 000–49 999 | 1414 | 1.1 | 0.62 [0.25, 1.53] | 0.63 [0.26, 1.53] |
| £13 500–24 999 | 1166 | 0.6 | 0.38 [0.13, 1.04] | 0.36 [0.13, 1.00] |
| up to £13 499 | 1674 | 1.1 | 0.61 [0.25, 1.48] | 0.58 [0.24, 1.40] |
| Missing | 6745 | - | - | - |
| CI, confidence interval. OR, odds ratio. OR_adj_, odds ratio adjusted for age, gender, survey year and level of tobacco addiction. SEP, socioeconomic position.  ^a^ Unweighted sample size.  ^b^ weighted dataset (refer to supplementary Table S32 for unweighted dataset percentages and effect estimates). | | | | |

## Table S32: Use of alternative treatments by socioeconomic position (unweighted dataset)

| **SEP indicator** | **N** | **Percentage of respondents who used the cessation aid by SES subcategories, %** | **OR [95% CI]** | **OR_adj_ [95%CI]** |
| --- | --- | --- | --- | --- |
| ***Occupational social grade*** |  |  |  |  |
| AB | 1738 | 1.4 | *ref* | *ref* |
| C1 | 3943 | 1.1 | 0.79 [0.49, 1.31] | 0.97 [0.60, 1.63] |
| C2 | 2456 | 0.9 | 0.65 [0.36, 1.15] | 0.70 [0.39, 1.25] |
| D | 1835 | 0.9 | 0.64 [0.34, 1.18] | 0.78 [0.41, 1.44] |
| E | 1767 | 0.5 | 0.35 [0.15, 0.73] | 0.31 [0.13, 0.66] |
| ***Employment status*** |  |  |  |  |
| Paid work | 6792 | 1.1 | *ref* | *ref* |
| Student | 749 | 0.8 | 0.72 [0.28, 1.53] | 2.21 [0.78, 5.34] |
| Not in paid work | 2832 | 0.7 | 0.64 [0.38, 1.02] | 0.48 [0.27, 0.81] |
| Retired | 1349 | 1.3 | 1.21 [0.70, 1.99] | 0.47 [0.19, 1.12] |
| Missing | 17 | - | - | - |
| ***Housing tenure*** |  |  |  |  |
| Owner occupied | 3213 | 1.4 | *ref* | *ref* |
| Private rented | 2437 | 0.7 | 0.46 [0.25, 0.79] | 0.67 [0.35, 1.22] |
| Social rented | 2578 | 0.7 | 0.51 [0.29, 0.86] | 0.53 [0.30, 0.92] |
| Other^a^ | 117 | 0.9 | 0.59 [0.03, 2.75] | 0.84 [0.04, 4.14] |
| Missing | 3394 | - | - | - |
| ***Education*** |  |  |  |  |
| University degree | 2931 | 1.1 | *ref* | *ref* |
| A-level/equivalent | 2771 | 1.1 | 1.02 [0.62, 1.70] | 1.24 [0.75, 2.07] |
| GCSE/Vocational | 3689 | 1.0 | 0.92 [0.57, 1.50] | 0.87 [0.53, 1.44] |
| No post-16 qualification | 1528 | 0.9 | 0.87 [0.44, 1.60] | 0.61 [0.30, 1.17] |
| Other/still studying | 736 | 1.0 | 0.90 [0.36, 1.93] | 0.91 [0.37, 1.96] |
| Missing | 84 | - | - | - |
| ***Household income*** |  |  |  |  |
| £50 000+ | 740 | 1.4 | *ref* | *ref* |
| £25 000–49 999 | 1414 | 1.1 | 0.78 [0.35, 1.81] | 0.78 [0.34, 1.84] |
| £13 500–24 999 | 1166 | 0.6 | 0.44 [0.16, 1.15] | 0.41 [0.14, 1.10] |
| up to £13 499 | 1674 | 0.8 | 0.62 [0.27, 1.43] | 0.56 [0.24, 1.38] |
| Missing | 6745 |  | - | - |
| CI, confidence interval. OR, odds ratio. OR_adj_, odds ratio adjusted for age, gender, survey year and level of tobacco addiction. SEP, socioeconomic position.  - not applicable | | | | |

## Figure S1: Direction of effect plot for cessation aids by each indicator of socioeconomic position and subcategories


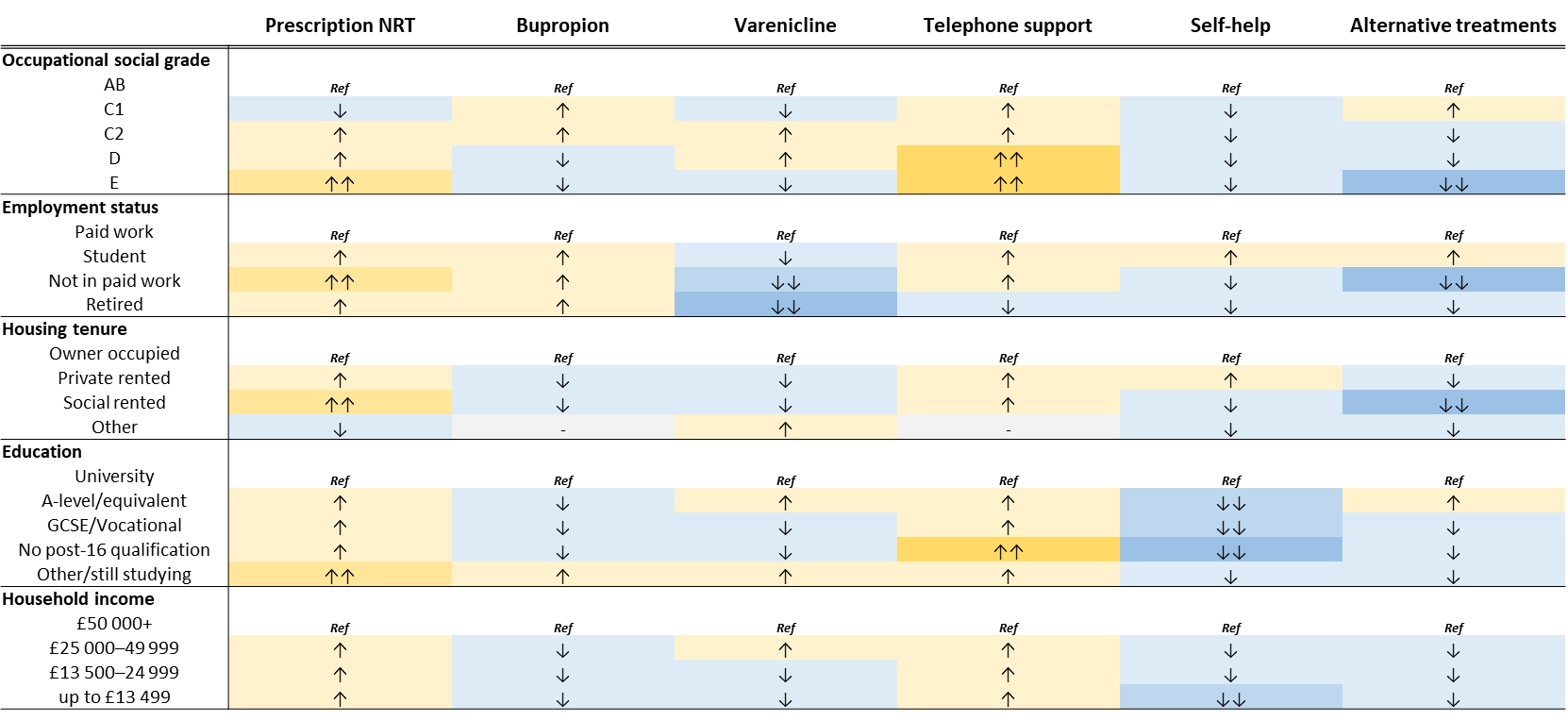


| **Legend** | |
| --- | --- |
| **↑↑** | Statistically significant greater odds for each outcome in the lower subcategories relative to highest subcategory of socioeconomic position for each indicator (point estimate OR ≥ 3.0) |
| **↑↑** | Statistically significantly greater odds/effect for each outcome in the lower subcategories relative to highest subcategory of socioeconomic position for each indicator (point estimate OR ≥ 2.0 to < 3.0) |
| **↑↑** | Statistically significantly greater odds/effect for each outcome in the lower subcategories relative to highest subcategory of socioeconomic position for each indicator (point estimate OR ≥ 1.0 to < 2.0) |
| **↑** | Direction of effect favours greater odds for each outcome in the lower relative to highest subcategories of socioeconomic position for each indicator; however, CI include the possibility of no difference (point estimate OR ≥ 1.0, but CI includes no statistically significant difference (lower bound of 95% CI < 1.0)) |
| **↓** | Direction of effect favours lower odds for each outcome in the lower relative to highest subcategories of socioeconomic position for each indicator; however, CI include the possibility of no difference (point estimate OR < 1.0, but 95% CI includes no statistically significant difference (upper bound of CI ≥ 1.0)) |
| **↓↓** | Statistically significantly lower odds/effect for each outcome in the lower relative to highest subcategories of socioeconomic position for each indicator (point estimate OR: ≥0.50 to <1.0) |
| **↓↓** | Statistically significantly lower odds/effect for each outcome in the lower relative to highest subcategories of socioeconomic position for each indicator (point estimate OR: ≥0.33 to <0.5) |
| **↓↓** | Statistically significantly lower odds/effect for each outcome in the lower relative to highest subcategories of socioeconomic position for each indicator (point estimate OR ≤ 0.33) |
| **-** | Reliable estimates could not be calculated due to small samples |

**Abbreviations: NRT:** Nicotine replacement therapy

Socioeconomic position (ordered highest to lowest socioeconomic subcategories):

- ***Occupational social grade:*** AB (higher managerial, administrative or professional); C1 (supervisory or clerical and junior managerial, administrative or professional); C2 (Skilled manual workers); D (Semi-skilled and unskilled manual workers), E (Casual or lowest grade workers, pensioners, and others who depend on the welfare state for their income).
- ***Employment status:*** paid work; student; not in paid work; retired.
- ***Housing tenure***: owner occupied; private rented (renting from private landlord); social rented (rented from local authority/housing association; other.
- ***Educational level:*** University degree, A-level or equivalent (high school senior); General Certificate of Secondary Education (GCSE)/O-level/CSE (high school sophomore) or vocational qualification (high school senior); No post-16 formal qualifications; Other or still studying.
- ***Annual household income:*** £50,000+; £25,000–49,999; £13,500–24,999; up to £13,499.

# Quitting success

## Research question 5

Among past-year smokers who made a quit attempt, to what extent does the use of cessation aids and quitting success differ by socioeconomic position after adjusting for age, gender, survey year and level of tobacco addiction?

## Table S33: Quitting success by socioeconomic position among past-year smokers who made a quit attempt

| **SEP Indicator** | **N^a^** | **Percentage of respondents who successfully quit by SES subcategories^b^** | **OR [95%CI]^b^** | **OR_adj_ [95%CI]^b^** |
| --- | --- | --- | --- | --- |
| ***Occupational social grade*** |  |  |  |  |
| AB | 1738 | 8.4 | *ref* | *ref* |
| C1 | 3943 | 8.2 | 1.02 [0.89, 1.18] | 1.05 [0.90, 1.23] |
| C2 | 2456 | 6.8 | 0.87 [0.74, 1.02] | 0.97 [0.82, 1.16] |
| D | 1835 | 6.0 | 0.72 [0.60, 0.86] | 0.83 [0.68, 1.00] |
| E | 1767 | 5.2 | 0.64 [0.53, 0.77] | 0.94 [0.76, 1.15] |
|  |  |  |  |  |
| ***Employment status*** |  |  |  |  |
| Paid work | 6792 | 7.6 | *ref* | *ref* |
| Student | 749 | 8.5 | 1.00 [0.82, 1.23] | 0.91 [0.73, 1.15] |
| Not in paid work | 2832 | 6.0 | 0.73 [0.64, 0.82] | 0.94 [0.82, 1.09] |
| Retired | 1349 | 5.4 | 0.98 [0.84, 1.14] | 1.14 [0.86, 1.52] |
| Missing | 17 | 1.4 | - | - |
|  |  |  |  |  |
| ***Housing tenure*** |  |  |  |  |
| Owner occupied | 3213 | 7.1 | *ref* | *ref* |
| Private rented | 2437 | 6.0 | 0.79 [0.68, 0.91] | 0.82 [0.70, 0.97] |
| Social rented | 2578 | 5.0 | 0.60 [0.52, 0.70] | 0.72 [0.61, 0.84] |
| Other | 117 | 8.4 | 0.94 [0.58, 1.51] | 0.79 [0.49, 1.27] |
| Missing | 3394 | 9.0 | - | - |
|  |  |  |  |  |
| ***Education*** |  |  |  |  |
| University degree | 2931 | 9.0 | *ref* | *ref* |
| A-level/equivalent | 2771 | 8.6 | 0.96 [0.84, 1.10] | 1.17 [1.01, 1.36] |
| GCSE/Vocational | 3689 | 5.9 | 0.68 [0.60, 0.78] | 0.89 [0.77, 1.03] |
| No post-16 qualification | 1528 | 4.4 | 0.62 [0.52, 0.74] | 0.84 [0.69, 1.03] |
| Other/still studying | 736 | 7.2 | 0.98 [0.79, 1.21] | 1.16 [0.92, 1.46] |
| Missing | 84 | 3.5 | - | - |
|  |  |  |  |  |
| ***Household income*** |  |  |  |  |
| £50 000+ | 740 | 8.7 | *ref* | *Ref* |
| £25 000–49 999 | 1414 | 7.5 | 1.02 [0.81, 1.28] | 1.03 [0.80, 1.33] |
| £13 500–24 999 | 1166 | 5.8 | 0.72 [0.56, 0.93] | 0.85 [0.65, 1.11] |
| up to £13 499 | 1674 | 5.4 | 0.72 [0.57, 0.91] | 0.92 [0.71, 1.19] |
| Missing | 6745 | 7.3 | - | - |
| CI, confidence interval. OR, odds ratio. OR_adj_, odds ratio adjusted for age, gender, survey year and level of tobacco addiction. SEP, socioeconomic position.  ^a^ Unweighted sample size.  ^b^ weighted dataset (refer to supplementary Table S34 for unweighted dataset percentages and effect estimates). | | | | |

## Table S34: Quitting success by socioeconomic position (unweighted dataset)

| **SEP Indicator** | **N** | **Percentage of respondents who successfully quit by SES subcategories, %** | **OR [95%CI]** | **OR_adj_ [95%CI]** |
| --- | --- | --- | --- | --- |
| ***Occupational social grade*** |  |  |  |  |
| AB | 1738 | 8.2 | *ref* | *ref* |
| C1 | 3943 | 8.1 | 1.02 [0.89, 1.17] | 1.04 [0.86, 1.25] |
| C2 | 2456 | 6.4 | 0.83 [0.71, 0.96] | 0.94 [0.76, 1.15] |
| D | 1835 | 5.6 | 0.69 [0.58, 0.81] | 0.83 [0.66, 1.04] |
| E | 1767 | 4.8 | 0.60 [0.51, 0.71] | 0.88 [0.69, 1.11] |
|  |  |  |  |  |
| ***Employment status*** |  |  |  |  |
| Paid work | 6792 | 7.6 | *ref* | *ref* |
| Student | 749 | 7.9 | 0.930.77, 1.12] | 0.87 [0.66, 1.15] |
| Not in paid work | 2832 | 5.7 | 0.69 [0.62, 0.78] | 0.93 [0.79, 1.10] |
| Retired | 1349 | 5.2 | 0.93 [0.80, 1.07] | 1.09 [0.77, 1.54] |
| Missing | 17 | 1.5 | - | - |
|  |  |  |  |  |
| ***Housing tenure*** |  |  |  |  |
| Owner occupied | 3213 | 7.0 | *ref* | *ref* |
| Private rented | 2437 | 5.9 | 0.77 [0.67, 0.88] | 0.80 [0.66, 0.96] |
| Social rented | 2578 | 4.7 | 0.56 [0.49, 0.64] | 0.67 [0.56, 0.81] |
| Other | 117 | 8.6 | 0.98 [0.61, 1.51] | 0.82 [0.44, 1.45] |
| Missing | 3394 | 9.1 | - | - |
|  |  |  |  |  |
| ***Education*** |  |  |  |  |
| University degree | 2931 | 8.8 | *ref* | *ref* |
| A-level/equivalent | 2771 | 8.3 | 0.94 [0.83, 1.07] | 1.17 [0.99, 1.40] |
| GCSE/Vocational | 3689 | 5.9 | 0.69 [0.61, 0.77] | 0.92 [0.78, 1.09] |
| No post-16 qualification | 736 | 4.2 | 0.60 [0.51, 0.70] | 0.85 [0.67, 1.07] |
| Other/still studying | 1528 | 6.7 | 0.93 [0.76, 1.12] | 1.13 [0.86, 1.48] |
| Missing | 84 | 3.4 | - | - |
|  |  |  |  |  |
| ***Household income*** |  |  |  |  |
| £50 000+ | 740 | 8.7 | *ref* | *ref* |
| £25 000–49 999 | 1414 | 7.2 | 0.95 [0.77, 1.19] | 1.00 [0.75, 1.33] |
| £13 500–24 999 | 1166 | 5.4 | 0.67 [0.53, 0.85] | 0.79 [0.58, 1.09] |
| up to £13 499 | 1674 | 5.2 | 0.68 [0.54, 0.85] | 0.86 [0.64, 1.16] |
| Missing | 6745 | 7.2 | - | - |
| CI, confidence interval. OR, odds ratio. OR_adj_, odds ratio adjusted for age, gender, survey year and level of tobacco addiction. SEP, socioeconomic position.  - not applicable | | | | |

# Protocol deviations

- We treated ‘level of tobacco addiction’ as a continuous variable as the response options could be viewed to increase by a similar about. As such, we used linear regression to calculate β values rather than OR effect estimates, with 95% CI.

**Protocol deviations from Final version 1 (prior to analysis)**

- Estimates of association for all outcome variables will be calculated using both weighted and unweighted data, with unweighted data reported in supplementary files.
- Research question 4 considers the extent to which the rate of past-year quit attempts differs by socioeconomic position amongst past year smokers, after adjusting for age, gender, survey year and motivation to quit. Given this question includes all past-year smokers (inclusive of respondents who had stopped smoking completely in the last year), respondents who stopped smoking in the last year would have missing data on motivation to quit, so this was removed as a covariate.
